# Comparative transcriptome analysis reveals K^+^ transporter gene contributing to salt tolerance in eggplant

**DOI:** 10.1186/s12870-019-1663-8

**Published:** 2019-02-11

**Authors:** Jing Li, Zhen Gao, Lu Zhou, Linzhi Li, Junhao Zhang, Yang Liu, Huoying Chen

**Affiliations:** School of Agriculture and Biology, Shanghai Jiao Tong University, 800 Dongchuan Road, Minhang District, Shanghai, 200240 China

**Keywords:** Eggplant (*Solanum melongena* L.), Salt stress, Comparative-transcriptome, Genotype-specific expression, *SmAKT1*

## Abstract

**Background:**

Soil salinization is one of the most crucial abiotic stresses that limit the growth and production of eggplant. The existing researches in eggplant were mostly focused on salt-induced morphological, biochemical and physiological changes, with only limited works centered on salt-response genes in eggplant at the transcriptomic level.

**Results:**

Our preliminary work found that Zhusiqie (No.118) is salt-tolerant and Hongqie (No.30) is salt-sensitive. Consequently, they were re-named as ST118 and SS30, respectively. ST118 showed less damaged on growth and higher K^+^/Na^+^ ratios in leaves than SS30. Comparative-transcriptome analysis was used as a powerful approach to understand the salt-response mechanisms in the leaves and roots of SS30 and ST118. And it revealed that genotype-specific and organ-specific manners exist in eggplant in response to salt stress. Strikingly, the genotype-specific differentially expressed genes (DEGs) in ST118 were considered crucial to its higher salt-tolerance, because the expression patterns of common DEGs in the leaves/roots of the two eggplant genotypes were almost the same. Among them, some transcription factors have been reported to be in response to elevated external salinity, including the members of C2C2-CO-like, WRKY, MYB and NAC family. In addition, the *AKT1*, *KAT1* and *SOS1* were up-regulated only in the leaves of ST118. Furthermore, the complementation assays demonstrated that the salt-tolerances of both yeast and Arabidopsis *akt1* mutants were enhanced by heterologous expression of *SmAKT1*.

**Conclusion:**

The comparative-transcriptome analysis indicated that the salt-tolerance can be increased by higher transcript level of some genotype-specific genes. This work revealed that eggplants seem to be more inclined to absorb K^+^ rather than to exclude Na^+^ under salt stress conditions because seven K^+^ transporters were significantly up-regulated, while only one Na^+^ transporter was similarly regulated. Finally, the complementation assays of *SmAKT1*, which is genotype-specific up-regulated in ST118, suggest that the other TFs and K^+^ transport genes were worthy of future investigation for their functions in salinity tolerance.

**Electronic supplementary material:**

The online version of this article (10.1186/s12870-019-1663-8) contains supplementary material, which is available to authorized users.

## Background

Soil salinity is one of the most important factors that limit plant growth, development, and productivity. According to the data from the FAO (Food and Agriculture Organization of the United Nations), food production should be increased by 70% in the world to meet the requirement of increasing population (http://www.fao.org/documents/card/en/c/a2128b09-361c-5468-9d93-2189cc430234/). In order to develop and utilize the salinized soil as much as possible, it is necessary to understand the salt-response mechanisms of crop plants.

To cope with salt stress, plants developed various protective mechanisms from the physiological and biochemical to the cellular and molecular level. On the molecular level, genes functioning in stress signaling, transcription regulation, ion transport and biosynthesis of specific metabolites are involved in responding to salt stress [1–4]. Transcription factors (TFs) involved in the regulation of salt-response could be activated by multiple signal transduction pathways in plants, such as the ABA-mediated signal [1, 5, 6]. Previous studies reported that the members of TF family genes were differentially expressed in response to elevated external salinity [7], including the APETALA2/ETHYLENE RESPONSE FACTOR (AP2/EREBP) [8], basic leucine zipper (bZIP) [6, 9], NAC [10, 11], basic helix–loop–helix (bHLH) [12], MYB [13–15] and WRKY [16, 17] gene families. In turn, these TFs could amplify the signals for gene regulation and promote the protective mechanisms in plants.

The major damage caused by excess salt was ion toxicity (mainly Na^+^) except water deficiency that is different from drought stress. The salt-overly-sensitive (SOS) signal transduction pathway has been described as crucial for cellular Na^+^ detoxification and maintaining intracellular ion homeostasis in plants [18–20]. However, excessive accumulation of Na^+^ under salt stress would be accompanied by K^+^ deficiency. Because of the similarity in physicochemical properties between Na^+^ and K^+^ (i.e. ionic radius and ion hydration energy), the root cells absorbed excessive Na^+^ instead of K^+^ under the saline soil [21], and ion homeostasis in plant cells could be destructed. K^+^ is one of the most important elements that is required by the key metabolic processes in the cytoplasm, including enzymatic reactions, protein synthesis, and ribosome functions [22]. Thus, K^+^/Na^+^ ratio is likely to be one of the key determinants of plant salt tolerance [22] and maintaining a high cytosolic K^+^/Na^+^ ratio is very important [23].

Eggplant (*Solanum melongena* L.) is an important greenhouse crop for out of season production and cultivated on more than 1.5Mha in the world [24]. Eggplant is considered as moderately sensitive to salinity with a very low threshold value [25, 26]. The existing researches in eggplant were focused on salt-induced morphological, biochemical and physiological changes [27–30]. However, there was limited work on salt-response genes in eggplant at the transcription level. Comparative genome and transcriptome have been extensively used as a powerful approach for discovering the genetic information involved in stress tolerance [31–33]. A number of transcriptomic comparisons have been done between salt-sensitive and salt-tolerant genotypes of plant species, such as Arabidopsis [34], *Oryza sativa* [3] and tomato [35]. Here, comparative transcriptome was used for the first time to explore the molecular mechanisms of salinity tolerance in eggplant.

In this work, the leaves and roots of two eggplant genotypes were exposed to salt-tolerant and comparative-transcriptome analysis under salt conditions. We successfully identified several TFs and ion transporters which might be crucial for the salt-tolerant eggplant genotype ST118 under salt conditions. In particular, a differentially expressed ion transporter was identified and functional verified which is potentially associated with eggplant responses and adaptability to salt conditions.

## Methods

### Plant material and growth conditions

Uniformly germinated eggplant seeds were selected and transplanted into growing trays with vermiculite and kept in growth chamber with 16/8 h light/dark photoperiod at 25/16 °C, respectively. About 1-months-old eggplant plants with four-true-leaves were treated with 200 mM NaCl. Roots and leaves for RNA extraction and ion content measurement were harvested at 0, 6, 12, 24, 48, 72, 168 h (7d) and 23d after stress treatments, respectively. After salt stress treatment for 23d, the phenotypic and physiological characteristics were inspected and measured. The samples at all the time points were used for ion content analysis. Based on the ion content difference between the two eggplant genotypes, samples collected at 0 h and 12 h were chosen for transcriptomic analysis.

The Arabidopsis (*Arabidopsis thaliana*) wild-type, mutant and transgenic plants used in this study were Columbia-0 ecotype (Col). The Arabidopsis seeds were germinated on Murashige and Skoog (MS) medium containing 0.8% (*w*/*v*) agar and 3% sucrose at 4 °C for 3 days. Then plates were incubated in a controlled-environment growth chamber. 3 days later, uniformly germinated seeds were chosen for low K^+^ or salt stress tests.

### Ion content measurement

All the samples were dried at 105 °C for 30 min, and then kept at 75 °C for 4 days. The grinded samples were digested in 20 ml HNO_3_, then added 5 mL HClO_4_ at room temperature. After overnight digestion, HNO_3_ and HClO_4_ were removed by heating. The digested samples were diluted with ddH_2_O. The Na^+^ and K^+^ contents were then measured by inductively coupled plasma optical (ICP-AES, iCAP7600).

### RNA extraction, library construction and illumina sequencing

Total RNA was extracted by the MiniBEST Universal RNA Extraction Kit (TaKaRa) according to the manufacturer’s instructions. The total RNA sample quality control (QC), library construction and sequencing on BGISEQ-500 was performed at Beijing Genomics Institute (BGI). The Agilent 2100 Bio analyzer (Agilent RNA 6000 Nano Kit) was used to do the total RNA sample QC, including RNA concentration, RIN value, 28S/18S and the fragment length distribution. The mRNA was enriched by magnetic beads with Oligo (dT) and then fragment the RNA and reverse transcription to double-strand cDNA (dscDNA) by N6 random primer. The synthesized cDNA was subjected to end-repair and then was 3′ adenylated. Adaptors were ligated to the ends of these 3′ adenylated cDNA fragments. The ligation products were purified and PCR amplification was performed to enrich the purified cDNA template using PCR primer. Lastly, the PCR products were denatured by heat and the single strand DNA was cyclized by splint oligo and DNA ligase. Then, the libraries were used for sequencing with the sequencing platform BGISEQ-500 (BGI), and the products were called as ‘raw reads’. All the generated raw sequencing reads were filtered to remove the low quality reads by the software SOAPnuke (BGI). After filteration, the remaining reads are called ‘Clean Reads’ and stored in FASTQ format.

### Bioinformatics analysis

After QC analysis, the clean reads were assembled into Unigenes and mapped to the eggplant genome sequences (http://eggplant.kazusa.or.jp/) [36] by HISAT (Hierarchical Indexing for Spliced Alignment of Transcripts) [37]. The gene expression level was calculated with RSEM [38]. Pearson’s correlation was exploited to calculate the relevance between all samples [39]. The differentially expressed genes (DEGs) were detected with DEGseq [40], which is based on the Poisson distribution. Combining the strategies described by Y Benjamini and Y Hochberg [41] and JD Storey and R Tibshirani [42], the *P*-values was adjusted as Q-values. And the threshold of Q-values ≤0.001 and an absolute Log2Ratio value ≥1 among the three biological replicates were used to determine whether a gene was DEG. The sequences of DEGs were compared with the NCBI non-redundant (Nr) database to identify and annotate the obtained DEGs using Blast software [43, 44].

Gene ontology (GO) functional classification of the identified DEGs was performed using Blast2GO [45]. GO enrichment analysis of the DEGs was conducted according to the information from GO databases (http://wego.genomics.org.cn/). Then we calculate the false discovery rate (FDR) for each *p*-value, in general, the terms which FDR ≤ 0.01 are defined as significant enriched. As for transcription factor prediction, getorf was used to find ORF of each DEG and then ORF was aligned to TF domains (from PlntfDB) using hmmsearch [46].

### Quantitative real-time PCR analysis

Total RNA was extracted by the MiniBEST Universal RNA Extraction Kit (TaKaRa). 500 ng RNA was transcribed into cDNA with the PrimeScript™ RT Master Mix (Perfect Real Time) (Takara). According to the manufacturer’s instructions of SYBR® Premix Ex Taq™ II (Tli RNaseH Plus) (Takara), qRT-PCR was performed on CFX Real Time PCR Detection System (BioRAD) using the following procedure: 95 °C for 30 s, followed by 40 cycles of 95 °C for 5 s and 60 °C for 30 s. The *Smactin* (*Sme2.5_00072.1_g00003.1*) from eggplant and *AtACT2* (AT3G18780.1) were amplified in parallel as internal reference genes, respectively. The relative expression levels of the amplified products were analyzed using the comparative CT method based on CT values [47]. All primers used in this study are listed in Additional file 1.

### Analysis of the protein structure

The protein sequences of AKT1 from 9 plant species were searched in the Pfam database (http://pfam.xfam.org/) and NCBI database (https://blast.ncbi.nlm.nih.gov/Blast.cgi). Then the sequences of each putative conserved domains were obtained using ClustalX (version 1.83) [48] and WebLogo 3 (http://weblogo.threeplusone.com/create.cgi). For phylogenetic analysis, ClustalX (version 1.83) and MEGA 6.0 [49] programs were used to construct neighbor-joining (NJ) tree with the following parameters: poission model, complete deletion and bootstrap (1000 replicates; random seed).

### Yeast complementation

The coding sequences of *SmAKT1* and *AtAKT1* were constructed into pYES2.0 vector and transformed into the yeast strain R5421 (*trk1△ trk2△*), in which the two endogenous K^+^ transporter genes (*TRK1*, *2*) were deleted. The yeast complementation assay was done as described by J Li, et al. [50]. After 5 days, all the plates were examined and photographed. Three independent experiments were performed.

### Generation of Arabidopsis transgenic plants

Full-length coding sequence of *SmAKT1* was constructed into the overexpression pHB vector. The construct was transformed into Arabidopsis *akt1* mutant. The Arabidopsis was transformed by the floral dip method with *Agrobacterium* [51]. The T4 homozygous transgenic plants were used to examine the phenotype under low-K^+^ [50] or salt stress conditions. The expression of targeted genes in complementary plants was detected using qRT-PCR.

### Salt tolerance assays of transgenic Arabidopsis

The K^+^ deficiency assay was done as described previously [52]. The phenotype was observed after low-K^+^ treatment for 7 d. For salt tolerance assays of transgenic Arabidopsis, 3-week-old wild-type, mutant and complementary plants were subjected to 200 mM NaCl treatment three times a week. The rosette leaves and roots of each Arabidopsis lines were collected for genes expression analysis after NaCl treatment for 0, 12 h and 7 days.

### Chlorophyll a fluorescence

Chlorophyll a fluorescence of Arabidopsis leaves was determined with the pulse-amplitude-modulated chlorophyll fluorescence system (PAM; Heinz Walz, GmbH, Effeltrich, Germany). Plants were kept in darkness for 30 min to quantify photosystem II (PSII; Fv/Fm)) maximum efficiency using the saturation pulse method: Fv/Fm = (Fm – F0)/Fm [53, 54]. Data are the means of 6 replicates.

### Data analysis

All data were presented as means with standard errors. The data were analyzed using SPSS 17.0 by one-way analysis of variance (ANOVA). Significance statistical analysis was calculated by Duncan’s Multiple Range test at significance levels of *P* < 0.05 and *P* < 0.01.

## Results

### Effect of salt stress on two eggplant genotypes

**Fig. 1:**
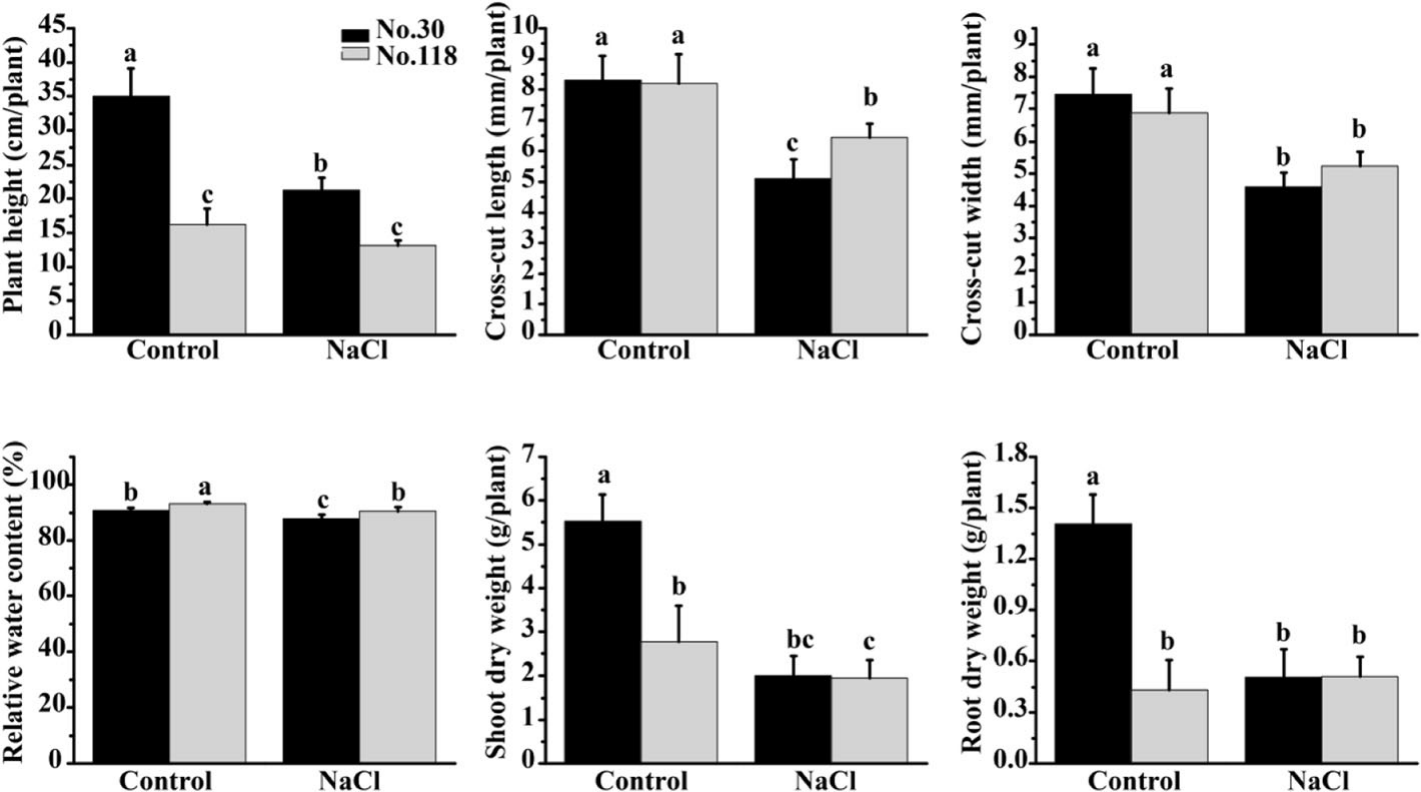
Morphological trait performance of two eggplant genotypes measured under control and salt conditions. The cross-cut length and width represent stem development. Bars represent means ± SD of three biological replicates. Columns with different letters indicate significant differences at *P* < 0.05 (Duncan’s test)

We investigated the salt tolerances of two eggplant genotypes, Hongqie (No.30) and Zhusiqie (No.118), four leaf-stage seedlings were irrigated with 200 mM NaCl. After 23 days, the phenotypic and physiological characteristics of No.30 and No.118 under salt conditions were compared with control. The salt-tolerance related trait values were dramatically reduced, and significant differences were detected between No.30 and No.118. As shown in Fig. 1, the plant height (PH), cross-cut length of stem, shoot dry weight (SDW) and root dry weight (RDW) were much reduced by salt stress in No.30 than No.118. However, no differences in the cross-cut width of stem and relative water content (RWC) between No.30 and No.118 were found.

**Fig. 2:**
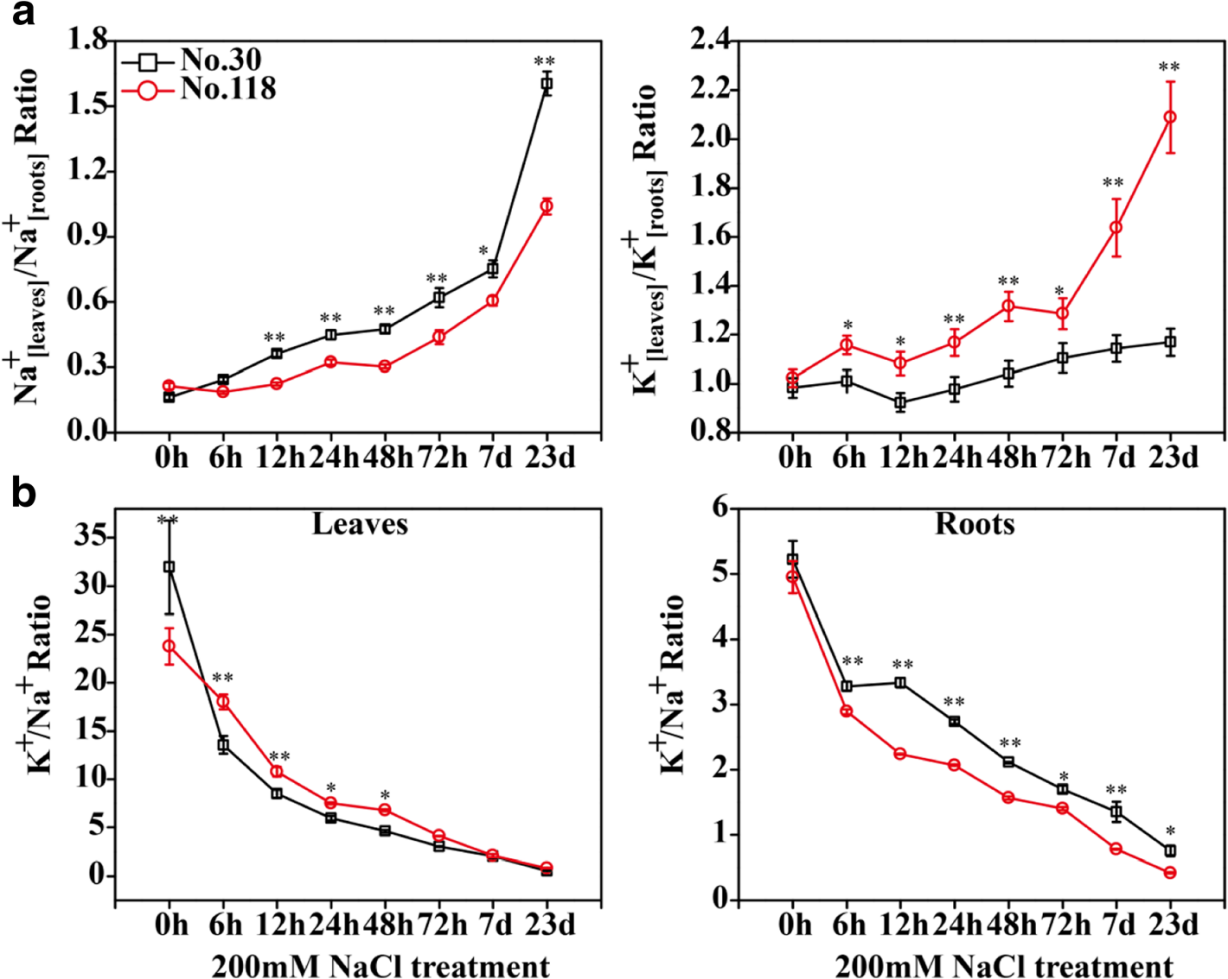
The distribution of Na^+^ and K^+^ and the change of K^+^/Na^+^ ratio in leaves/roots of two eggplant genotypes along with salt treatment. **a** The distribution of Na^+^ and K^+^ in leaves and roots. **b** The change of K^+^/Na^+^ ratio in leaves and roots. Three replicates were used in each time point, with three seedlings per replicate. Bars represent means ± SD of three biological replicates. Duncan’s Multiple Range test (*P < 0.05 and **P < 0.01) was used to analyze statistical significance

Furthermore, the concentration and distribution of Na^+^ and K^+^ were affected along with NaCl stress treatment for 0, 6, 12, 24, 48, 72, 168 h (7 days) and 23 days (Fig. 2 and Additional file 2). The Na^+^ concentrations increased significantly while K^+^ concentrations reduced in leaves and roots of both two eggplant genotypes (Additional file 2). As shown in Additional file 2, lower total K^+^ concentrations (total content covers leaves and roots) but higher total Na^+^ concentrations were found in No.118 than No.30, and this difference peaked after salt treatment for 12 h. However, the Na^+^_[leaves]_/Na^+^_[roots]_ ratio increased less and the K^+^_[leaves]_/K^+^_[roots]_ ratio increased more in No.118 than in No.30 (Fig. 2a). In addition, the K^+^/Na^+^ ratios were gradually reduced in both leaves and roots along with the salt treatment, while a higher decrease found in No.30 leaves and No.118 roots (Fig. 2b).

These results suggested that K^+^ in No.118 was preferentially translocated into leaves, resulting in a higher K^+^/Na^+^ ratios. They also indicated that No.30 is more salt-sensitive than No.118. Therefore, we named the two eggplant genotypes as SS30 and ST118, respectively. Taken all together, the 0 h and 12 h time points were chosen for exploring the distribution mechanism of K^+^ and Na^+^, which might be a crucial point to explain the salt-tolerant difference between the two eggplant genotypes.

### Identification of differentially expressed genes in SS30 and ST118 by RNA-seq

**Fig. 3:**
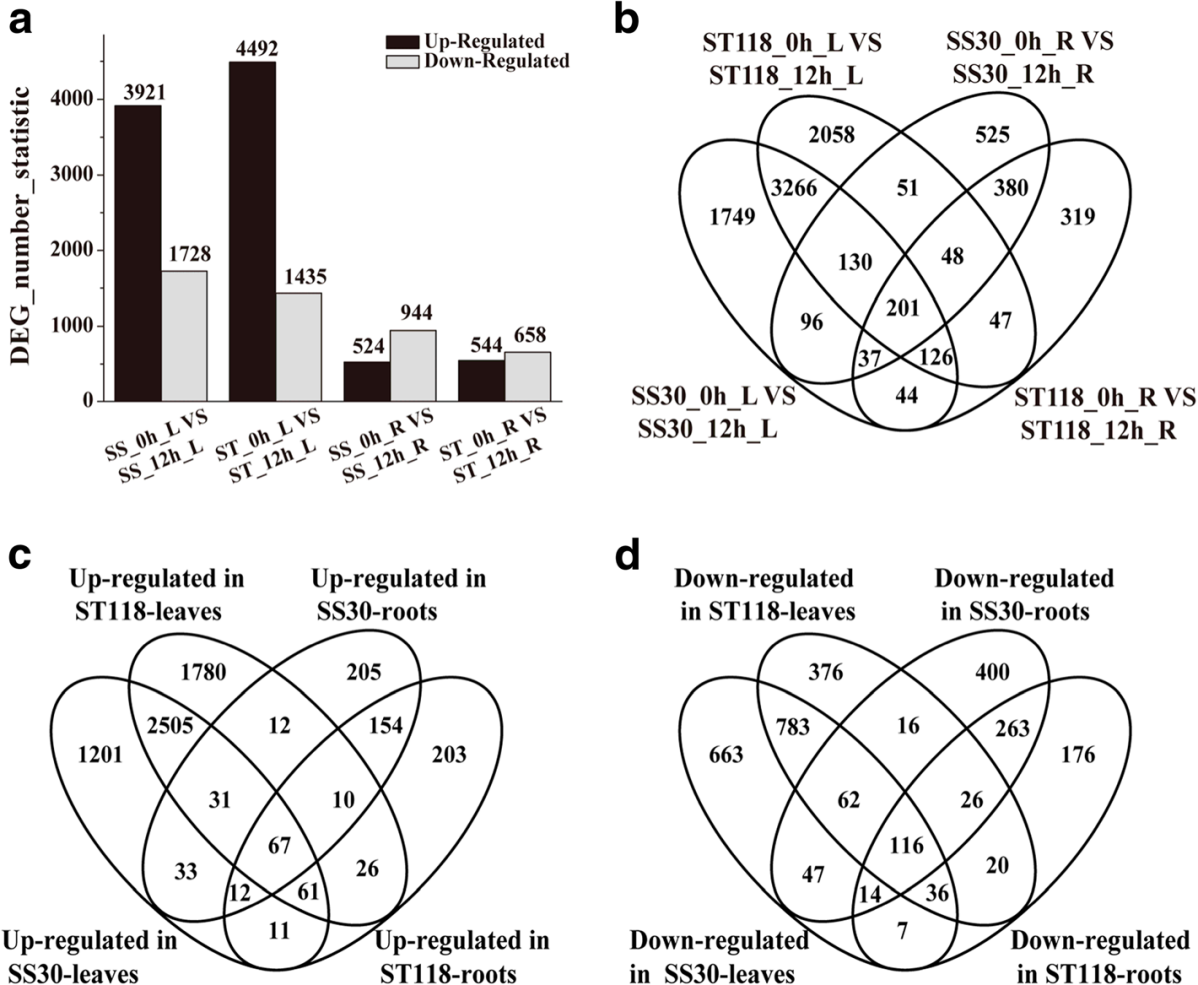
Overview and Venn diagrams of up- or down-regulated genes by salt stress in leaves and roots of both two eggplant materials at a level of ≥2-fold and adjusted P-value ≤0.001. **a** The total number of differentially expressed genes found in the leaves (L) and roots (R) of SS30 and ST118 in the comparison between salt-stressed (12 h) and non-stress treatments (0 h). **b** Four-way Venn diagram indicating that the DEGs were genotype-specific. The number of salt-up-regulated (**c**) and -down-regulated (**d**) genes found exclusively in the roots and leaves of two eggplant genotypes were analyzed

The leaves and roots were harvested from eggplants that have been treated with 200 mM NaCl for 0 h and 12 h, respectively. Using the BGISEQ-500 platform, an average about 24.11 M from 12 leaves samples and 23.77 M reads from 12 roots samples were generated, respectively (Additional file 3). In this project, the average mapping ratios with reference genome were 94.31% (leaves) and 89.85% (roots), the average mapping ratios with genes were 65.27% (leaves) and 58.47% (roots), and total of 50,956 (leaves) and 49,354 (roots) genes were detected. The differentially expressed genes (DEGs) were selected on the basis of DEGseq method with the following parameters: fold change ≥2 and adjusted *P*-value ≤0.001 [40]. A total of 5649 and 5927 DEGs were obtained in the leaves of SS30 and ST118 (Fig. 3a), while 1468 and 1202 DEGs were obtained in the roots of SS30 and ST118 (Fig. 3a). Subsequently, nine DEGs with different expression pattern were selected to validate the RNA-seq results by qRT-PCR (Additional file 4). Despite some differences, the general expression profiles were conserved between the RNA-seq and qRT-PCR data, which validates the former.

Venn analysis showed that the DEGs identified in both SS30 and ST118 have relatively same expression patterns except 62/3723 (62 out of 3723) in leaves and 4/ 666 in roots (Additional file 5). Among these DEGs, 2664/997 and 243/419 genes were commonly up−/down-regulated in the leaves and roots of both genotypes (Additional file 5). On the other side, only 67/116 genes were commonly up−/down-regulated in both the leaves and roots of the two eggplant genotypes under salt conditions (Fig. 3c, d), indicating that the organ-specific manner adapt to salt stress, observed in rice [2] and Arabidopsis [55], also exists in eggplant.

As shown in Fig. 3b, many DEGs identified under salt conditions were genotype-specific, suggesting that the genotype-specific DEGs might be contributed to the phenotypic differences in salt-tolerance between SS30 and ST118. The Venn diagram in Fig. 3c, d showed that 1201/663 and 205/400 genes were exclusively up−/down-regulated in the leaves and roots of SS30, while 1780/376 and 203/176 genes were exclusively up−/down-regulated in the leaves and roots of ST118. Since ST118 is more salt tolerant than SS30, more attention has been paid to the genotype-specific DEGs in ST118 in the following sections.

### Gene ontology analysis of DEGs

The functions of all the DEGs identified in this project were classified by the Gene Ontology (GO) assignments [56]. There were three GO categories including molecular function, biological process and cellular component in leaves and roots of both SS30 and ST118 (Additional file 6). The two largest subcategories found in the three GO categories were consistent, which were ‘metabolic process’ and ‘cellular process’ in the ‘biological process’ category, ‘cell’ and ‘cell part’ in the ‘cellular component’ category, and catalytic activity’ and ‘binding activity’ in the ‘molecular function’ category. Strikingly, the ‘response to stimulus’, ‘transporter activity’ and ‘transcription factor activity, protein binding’ were abundant GO terms.

**Fig. 4:**
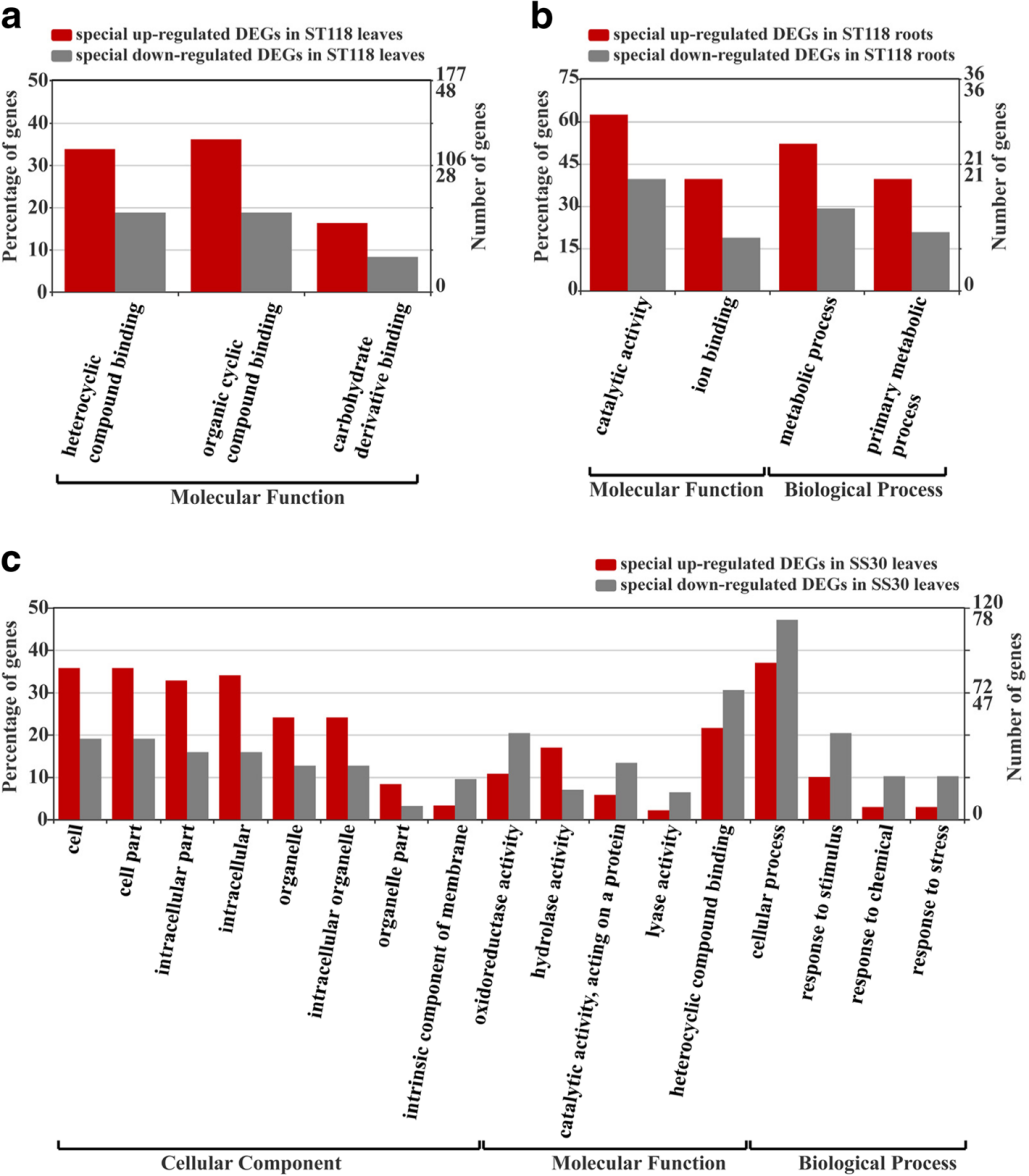
GO classification of the genotype-specific DEGs in the leaves/roots of SS30 or ST118. The left y-axis shows the percentages of genes identified, and the right y-axis shows the gene number. The genes were categorized according to the annotation of GO, and the number of every category is displayed based on biological process, cellular components, and molecular functions. The enriched GO terms were identified using a threshold of *P*-value < 0.05

GO terms enriched in the genotype-specific DEGs of SS30 or ST118 were identified using a threshold of *P*-value < 0.05. In the leaves of ST118, three GO terms were significantly enriched in the ‘molecular function’ category (Fig. 4a). Four GO terms were most abundant in the ‘molecular function’ and ‘biological process’ categories in the roots of ST118 (Fig. 4b). As for SS30, 17 GO terms were distributed in three GO categories including cellular component, molecular function and biological process in the leaves (Fig. 4c), but none GO terms were significantly enriched in the roots. Generally, the up-regulated genes enriched in both leaves and roots ST118 were much more than down-regulated genes, while opposite results showed in the molecular function and biological process categories of SS30. Compared with SS30 leaves, the genes related to ‘organic cyclic compound binding’ and ‘carbohydrate derivative binding’ activities were significantly enriched in ST118 leaves (Fig. 4a). In addition, genes related to ‘ion binding’ activity were exclusively enriched in the roots of ST118. These results suggested that genes with the binding sites for ion, inorganic or organic molecules might play important roles in response to salt stress.

### Differentially expressed transcription factors in SS30 and ST118 caused by salt stress

10 transcription factors (TFs) were found through the analysis of the genes related to ‘heterocyclic compound binding’, ‘organic cyclic compound binding’ and ‘carbohydrate derivative binding’ activities. Considering the crucial role of TFs in response to salt stress, we highlighted the analysis on the TFs that were identified as DEGs in leaves and roots of both SS30 and ST118.

In leaves, a total of 413 TFs were identified as DEGs. Among the 413 TFs, 201 TFs were identified as DEGs in both SS30 and ST118 (named as ST-SS-L-inter), while 120 and 92 TFs were specifically identified as DEGs in SS30 (named as SS30-L-Spe) and ST118 (named ST118-L-Spe), respectively (Additional file 7a). As shown in Additional file 7a, the highest rates of induction by salt stress were observed for genes belonging to the AP2/EREBP, MYB, bHLH, WRKY, NAC, ABI3/VP1, C3H, GRAS and C2C2-Dof families. Strikingly, AP2-EREBP and MYB super-families were the largest in ST118-L-Spe and ST-SS-L-inter, while the WRKY super-family was the largest in the SS30-L-Spe. Members of these identified TFs have been reported to be associated with salt stress responses [8, 17, 57, 58]. Subsequently, the 413 TFs were searched against the Stress Responsive Transcription Factor Database (STIDB) in Arabidopsis [58] for salt-responsive genes. 43 TFs were identified as salt-response genes, including 10 *MYB*s, 10 *NAC*s, 6 *WRKY*s, 5 *AP2-EREBP*s, 3 *C2C2-CO-like*s (*COL*), 3 *TAZ*s, 2 *bHLH*s, 2 *Tify*s, *C3H* and *G2-like* (Table 1). Generally, the expression patterns in majority of TFs were same and most of them were down-regulated by salt stress (Table 1). Strikingly, in ST-SS-L-inter, *Sme2.5_00556.1_g00019.1* annotated as *WRKY* was significantly up-regulated in ST118, but was significantly down-regulated in SS30. In addition, three TFs were slightly up-regulated in ST118 with 0.60~0.99 folds change, but significantly down-regulated in SS30 with − 3.10~ − 1.30 folds change (Table 1), including *Sme2.5_03951.1_g00007.1* (annotated as *MYB*), *Sme2.5_03886.1_g00002.1* (annotated as *NAC*) and *Sme2.5_04464.1_g00002.1* (annotated as *NAC*).

**Table 1:** The salt-responsive TFs identified by searching against the Stress Responsive Transcription Factor Database (STIDB) in Arabidopsis in the leaves and roots of the two eggplant materials. SS_0h_L/R-Expression: the mean expression level of each TF after NaCl treatment for 0 h in the leaves/roots of SS30; log2Ratio(s2/s1): Log2(folds of mean expression in two time points); q-value: corrected *p*-value [41, 42]; Tair10: gene ID in Arabidosis corresponding to the TFs in eggplant

	Gene ID	SS_0h_L-Expression	SS_12h_L-Expression	log2Ratio(SS_12h_L/SS_0h_L)	q-value	ST_0h_L-Expression	ST_12h_L-Expression	log2Ratio(ST_12h_L/ST_0h_L)	q-value	Tair10	TF family
ST-SS-L-inter	Sme2.5_05868.1_g00004.1	10,615	340	−4.90	0.000	9160	198	−5.51	0.000	AT5G17300.1	MYB
	Sme2.5_02956.1_g00004.1	1971	82	−4.52	0.000	1949	53	−5.18	0.000	AT5G17300.1	MYB
	Sme2.5_02470.1_g00007.1	69	2	−5.04	0.000	26	3	−3.09	0.000	AT1G69490.1	NAC
	Sme2.5_00096.1_g00018.1	270.17	21.3	−3.60	0.000	412.04	71.58	−2.50	0.000	AT5G57550.1	NAC
	Sme2.5_01620.1_g00005.1	797	204	−1.90	0.000	837	169	−2.29	0.000	AT4G37180.1	G2-like
	Sme2.5_08291.1_g00004.1	845	176	−2.19	0.000	728	161	−2.15	0.000	AT1G70000.2	MYB
	Sme2.5_03858.1_g00003.1	482	133	−1.79	0.000	538	162	− 1.71	0.000	AT1G13260.1	AP2-EREBP
	Sme2.5_00276.1_g00022.1	788	107	−2.81	0.000	759	229	−1.71	0.000	AT1G01720.1	NAC
	Sme2.5_11816.1_g00001.1	60.47	3.95	−3.87	0.000	41.65	12.75	−1.69	0.000	AT3G23250.1	MYB
	Sme2.5_01393.1_g00007.1	4149	1469	−1.43	0.000	3623	1217	−1.55	0.000	AT1G05690.1	TAZ
	Sme2.5_15135.1_g00001.1	1360	291	−2.16	0.000	609	209	−1.52	0.000	AT1G69490.1	NAC
	Sme2.5_06280.1_g00002.1	3950	1499	−1.33	0.000	4342	1544	−1.47	0.000	AT5G17300.1	MYB
	Sme2.5_06485.1_g00004.1	8176	3330	−1.23	0.000	8302	2979	−1.46	0.000	AT5G24930.1	C2C2-CO-like
	Sme2.5_01575.1_g00001.1	3303	881	−1.84	0.000	2935	1065	−1.44	0.000	AT3G49530.1	NAC
	Sme2.5_00014.1_g00027.1	526.44	234	−1.10	0.000	723.52	281	−1.34	0.000	AT1G06180.1	MYB
	Sme2.5_04750.1_g00001.1	694.26	114.48	−2.53	0.000	826.05	328.84	−1.31	0.000	AT4G25480.1	AP2-EREBP
	Sme2.5_07791.1_g00001.1	7184	3294	−1.06	0.000	7823	3284	−1.23	0.000	AT3G59060.4	bHLH
	Sme2.5_01200.1_g00003.1	215	51	−2.01	0.000	428	192	−1.13	0.000	AT1G19180.1	Tify
	Sme2.5_00556.1_g00019.1	57	6	−3.18	0.000	55	125	1.21	0.000	AT1G80840.1	WRKY
	Sme2.5_00912.1_g00004.1	380	800	1.14	0.000	369	965	1.41	0.000	AT3G47600.1	MYB
	Sme2.5_00183.1_g00008.1	1957.44	3883.65	1.06	0.000	1410.45	4358.31	1.65	0.000	AT5G58620.1	C3H
	Sme2.5_00196.1_g00009.1	28	4583	7.42	0.000	8	4224	9.07	0.000	AT3G07650.4	C2C2-CO-like
ST118-L-Spe	Sme2.5_00956.1_g00005.1	3381	2072	−0.64	0.000	4888	1138	−2.08	0.000	AT3G48360.1	TAZ
	Sme2.5_25982.1_g00001.1	3698	1883	−0.90	0.000	3651	1404	−1.36	0.000	AT5G57660.1	C2C2-CO-like
	Sme2.5_08533.1_g00002.1	36.34	19.06	−0.86	0.037	60.75	26.25	−1.19	0.000	AT3G23250.1	MYB
	Sme2.5_00641.1_g00007.1	157	157	0.07	0.488	295.19	129	−1.17	0.000	AT4G27410.2	NAC
	Sme2.5_06310.1_g00004.1	296.78	158.58	−0.84	0.000	292.67	129.1	−1.16	0.000	AT2G36800.1	WRKY
	Sme2.5_00332.1_g00002.1	8112.3	4172.0	−0.89	0.000	8253.0	3671.4	−1.15	0.000	AT1G19000.2	MYB
	Sme2.5_12868.1_g00001.1	6358	3737	−0.70	0.000	5384	2440	−1.12	0.000	AT3G16770.1	AP2-EREBP
	Sme2.5_00048.1_g00015.1	481	727	0.66	0.000	1070	2124	1.01	0.000	AT5G63160.1	TAZ
	Sme2.5_04588.1_g00001.1	200	213	0.16	0.240	137	333	1.30	0.000	AT4G28140.1	AP2-EREBP
	Sme2.5_00556.1_g00018.1	47	71	0.66	0.017	14	63	2.19	0.000	AT1G80840.1	WRKY
SS30-L-Spe	Sme2.5_29353.1_g00001.1	432.73	0	−9.69	0.000	24.3	19.19	−0.32	0.368	AT4G27410.3	NAC
	Sme2.5_03951.1_g00007.1	77	8	−3.20	0.000	59	96	0.72	0.003	AT3G23250.1	MYB
	Sme2.5_04750.1_g00004.1	112.81	13.96	−2.95	0.000	93.19	46.66	−0.98	0.000	AT4G25480.1	AP2-EREBP
	Sme2.5_03886.1_g00002.1	48	6	−2.93	0.000	32	63	1.00	0.001	AT2G43000.1	NAC
	Sme2.5_04190.1_g00001.1	110	16	−2.71	0.000	121	92	−0.37	0.064	AT1G80840.1	WRKY
	Sme2.5_01372.1_g00013.1	5293	1538	−1.71	0.000	4593	3439	−0.40	0.000	AT1G80840.1	WRKY
	Sme2.5_04464.1_g00002.1	55	22	−1.25	0.000	34	51	0.61	0.059	AT2G43000.1	NAC
	Sme2.5_02104.1_g00004.1	4983	2298	−1.05	0.000	3591	3810	0.11	0.002	AT1G32640.1	bHLH
	Sme2.5_04924.1_g00003.1	3418	1595	−1.03	0.000	3295	1821	−0.83	0.000	AT1G19180.1	Tify
	Sme2.5_04168.1_g00003.1	5649	2674	−1.01	0.000	3244	1645	−0.96	0.000	AT1G01720.1	NAC
	Sme2.5_15021.1_g00001.1	232	566	1.36	0.000	352	622	0.84	0.000	AT2G30590.1	WRKY
	GeneID	SS30_0h-Expression	SS30_12h-Expression	log2Ratio(SS30_12h/SS30_0h)	q-value(Storey et al. 2003)	ST118_0h-Expression	ST118_12h-Expression	log2Ratio(ST118_12h/ST118_0h)	q-value(Storey et al. 2003)	Tair 10	TF family
ST-SS-R-inter	Sme2.5_02956.1_g00004.1	1051	128	−3.04	0.000	1040	51	−4.43	0.000	AT5G17300.1	MYB
	Sme2.5_25982.1_g00001.1	1392	197	−2.82	0.000	1473	84	−4.21	0.000	AT5G57660.1	C2C2-CO-like
	Sme2.5_05868.1_g00004.1	937	123	−2.93	0.000	844	88	−3.34	0.000	AT5G17300.1	MYB
	Sme2.5_06280.1_g00002.1	2912	512	−2.51	0.000	2233	478	−2.30	0.000	AT5G17300.1	MYB
	Sme2.5_06485.1_g00004.1	969	401	−1.27	0.000	1205	372	−1.77	0.000	AT5G24930.1	C2C2-CO-like
	Sme2.5_07791.1_g00001.1	415	166	−1.32	0.000	310	148	−1.14	0.000	AT3G59060.4	bHLH
	Sme2.5_08000.1_g00008.1	140	392	1.49	0.000	151	571	1.84	0.000	AT3G22830.1	HSF
	Sme2.5_00196.1_g00009.1	72	794	3.46	0.000	30	915	4.85	0.000	AT3G07650.4	C2C2-CO-like
ST118-R-Spe	Sme2.5_00956.1_g00005.1	1900	1231	−0.62	0.000	1857	722	−1.44	0.000	AT3G48360.1	TAZ
	Sme2.5_08291.1_g00004.1	1321	684	−0.95	0.000	1487	702	−1.16	0.000	AT1G70000.2	MYB
	Sme2.5_00374.1_g00013.1	1098	606	−0.86	0.000	889	462	−1.02	0.000	AT1G78080.1	AP2-EREBP
	Sme2.5_06157.1_g00002.1	1512	2700	0.84	0.000	1314	3453	1.32	0.000	AT3G16770.1	AP2-EREBP
	Sme2.5_00048.1_g00015.1	3223	2556	−0.33	0.000	2486	6901	1.39	0.000	AT5G63160.1	TAZ
SS30-R-Spe	Sme2.5_29353.1_g00001.1	218.17	7.6	−4.84	0.000	0	5.73	3.44	0.061	AT4G27410.3	NAC
	Sme2.5_02470.1_g00007.1	160	20	−3.00	0.000	51	27	−1.00	0.009	AT1G69490.1	NAC
	Sme2.5_15135.1_g00001.1	48	12	−2.00	0.000	14	17	0.20	0.651	AT1G69490.1	NAC
	Sme2.5_04464.1_g00002.1	56	16	−1.81	0.000	27	17	−0.75	0.159	AT2G43000.1	NAC
	Sme2.5_03858.1_g00003.1	491	157	−1.64	0.000	350	301	−0.30	0.023	AT1G13260.1	AP2-EREBP
	Sme2.5_03434.1_g00003.1	77	26	−1.56	0.000	67	51	−0.47	0.139	AT2G31180.1	MYB
	Sme2.5_24078.1_g00001.1	4527	2049	−1.14	0.000	4664	2685	−0.87	0.000	AT5G67480.2	TAZ
	Sme2.5_09948.1_g00004.1	41.98	83.96	1.00	0.001	41.92	59.97	0.44	0.208	AT5G26660.1	MYB

In roots, 147 TFs were obtained including 58 ST-SS-R-inter TFs, 56 SS30-R-Spe and 33 ST118-R-Spe TFs (Additional file 7b), and the highest rates of TFs belong to AP2/EREBP, MYB and bHLH families. After searching against STIDB in Arabidopsis [58], 21 TFs were found to be salt-response genes (Table 1), including 6 *MYB*s, 4 *NAC*s, 3 *AP2-EREBP*s, 3 *COL*s, 3 *TAZ*s, *bHLH* and *HSF*. Among the 21 TFs, 11 TFs could also be identified as DEGs in leaves with the same expression pattern. Of the 11 TFs, one C2C2-CO-like family member was highly up-regulated by salt-stress with 9.1/3.5 and 7.4/4.9 folds in the leaves/roots of SS30 and ST118, respectively.

These results indicated that the basal salt-resistance mechanism was the same in eggplant varieties, but the specifically up-regulated TFs in SS118 might make a positive contribution to its salt-tolerance.

### Identification of the DEGs related to ion transport in SS30 and ST118 under salt condition

K^+^/Na^+^ ratio is one of the key determinants of plant salt tolerance, and significant difference was found between SS30 and ST118. Although the ‘transporter activity’ category was enriched in both SS30 and ST118, the number and the members of genes were different. Analysis of these genes involved in the ‘transporter activity’ category showed that 43 DEGs belonged to ST-SS-L-inter, while 24 and 16 DEGs belonged to ST118-L-Spe and SS30-L-Spe, respectively.

In the ST-SS-L-inter category, five DEGs were identified as K^+^ transporter or K^+^ channel protein compared with the NCBI non-redundant (Nr) database [43] and all of them were upregulated by salt stress (Table 2). Except the five genes encoding K^+^ transporters or K^+^ channel proteins, another K^+^ transporter gene (*AKT1*) and K^+^ channel gene (*KAT1*) were specifically up-regulated by salt stress in ST118. Strikingly, the ‘salt overly sensitive’ (*SOS1*) gene was exclusively up-regulated in ST118 but was slightly down-regulated in SS30 (Table 2). However, no more genes related to K^+^ and Na^+^ homeostasis were found in SS30-L-spe. The specifically up-regulated expression of *AKT1*, *KAT1* and *SOS1* in ST-118 during salinity stress would be expected to stabilize the K^+^/Na^+^ ratio in leaves (Fig. 2b).

**Table 2:** The different expressed genes related to K^+^ and Na^+^ transport in the leaves of the two eggplant materials

	Gene ID	SS_0h_L-Expression	SS_12h_L-Expression	log2Ratio(SS_12h_L/SS_0h_L)	q-value	ST_0h_L-Expression	ST_12h_L-Expression	log2Ratio(ST_12h_L/ST_0h_L)	q-value	Tair10	Transporter name
ST-SS-L-inter	Sme2.5_08678.1_g00002.1	260.9	560.85	1.17	0.000	212.94	457.93	1.13	0.000	AT2G26650.1	AKT1-like
	Sme2.5_09079.1_g00001.1	9.1	32.15	1.89	0.000	5.06	14.07	1.50	0.000	AT2G26650.1	AKT1
	Sme2.5_02726.1_g00002.1	306.99	698.29	1.25	0.000	344.47	971.36	1.52	0.000	AT2G40540.2	potassium transporter 2
	Sme2.5_00325.1_g00013.1	102.57	949.18	3.28	0.000	111.92	1011.9	3.20	0.000	AT5G55630.2	K+ channel protein
	Sme2.5_30443.1_g00001.1	60.36	729.87	3.66	0.000	31.83	1026.2	5.03	0.000	AT5G55630.2	K+ channel protein
ST118-L-spe	Sme2.5_00191.1_g00006.1	1541	2318	0.66	0.000	1140	2338	1.06	0.000	AT5G46240.1	KAT1
	Sme2.5_00439.1_g00001.1	836	1349	0.76	0.000	638	1452	1.21	0.000	AT2G26650.1	AKT1
	Sme2.5_05879.1_g00004.1	202	158	−0.29	0.069	69	169	1.31	0.000	AT2G01980.1	SOS1
	GeneID	SS_0h_R-Expression	SS_12h_R-Expression	log2Ratio(SS_12h_R/SS_0h_R)	q-value	ST_0h_R-Expression	ST_12h_R-Expression	log2Ratio(ST_12h_R/ST_0h_R)	q-value		
In Roots	Sme2.5_08678.1_g00002.1	390.71	354.69	−0.14	0.289	364.86	299.73	−0.36	0.004		
	Sme2.5_09079.1_g00001.1	64.29	44.31	−0.54	0.109	32.14	38.27	0.17	0.604		
	Sme2.5_02726.1_g00002.1	217.01	286.87	0.40	0.006	332.17	404.02	0.20	0.106		
	Sme2.5_00325.1_g00013.1	553.43	699.6	0.34	0.000	461.18	731.5	0.59	0.000		
	Sme2.5_30443.1_g00001.1	266.8	388.9	0.55	0.000	286.51	463.58	0.62	0.000		
	Sme2.5_00191.1_g00006.1	3	2	−0.58	0.642	3	4	0.34	0.683		
	Sme2.5_00439.1_g00001.1	3863	3152	−0.29	0.000	3710	3041	−0.36	0.000		
	Sme2.5_05879.1_g00004.1	238	287	0.27	0.069	242	305	0.26	0.080		

In the roots of both salt-tolerant and salt-sensitive eggplant varieties, none genes related to K^+^ and Na^+^ homeostasis was identified as DEG. Further analysis showed that the 8 ion transporter genes identified in leaves remained higher expression level in roots under both control and salt condition comparing with leaves, except for *Sme2.5_00191.1_g00006.1* (*KAT1*) and *Sme2.5_02726.1_g00002.1* (a K^+^ transporter gene) (Table 2). This might be the reason that salinity tolerance is more related to the fine tuning of the ion transporter genes rather than to significant up−/down-regulate these genes by salt stress in roots [3].

It is well known that SOS signaling pathway was the first demonstrated regulator in mediating Na^+^ extrusion in Arabidopsis and later in other plant species [23, 59–61]. Here, more genes closely related to K^+^ absorption than those related to Na^+^ extrusion were found to be up-regulated, indicating that K^+^ absorption is equally important with Na^+^ extrusion for maintaining K^+^ and Na^+^ homeostasis in plants under salt conditions.

### Functional characterization of *SmAKT1* in yeast and Arabidopsis under salt conditions

A series of studies showed that *AKT1* plays an important role on resisting low-K^+^ stress in plants [62–64]. However, the function of *AKT1* in eggplant under low K^+^-starvation and salt stress has not been report so far. The full-length amino acid sequences of the two identified AKT1s (*Sme2.5_09079.1_g00001.1* and *Sme2.5_00439.1_g00001.1*) in eggplant together with AKT1 from the other nine plant species were aligned separately and a bootstrapped consensus neighbor-joining (NJ) tree was inferred for SmAKT1 (Additional file 8). As shown in Additional file 8, Sme2.5_00439.1_g00001.1 had the highest degree of similarity with AKT1s from the other plant species. In addition, the typically conserved domains of AKT1 were also found in an 884 amino acid polypeptide of Sme2.5_00439.1_g00001.1 (Additional file 9). Taken together, the Sme2.5_00439.1_g00001.1 could be named as SmAKT1.

**Fig. 5:**
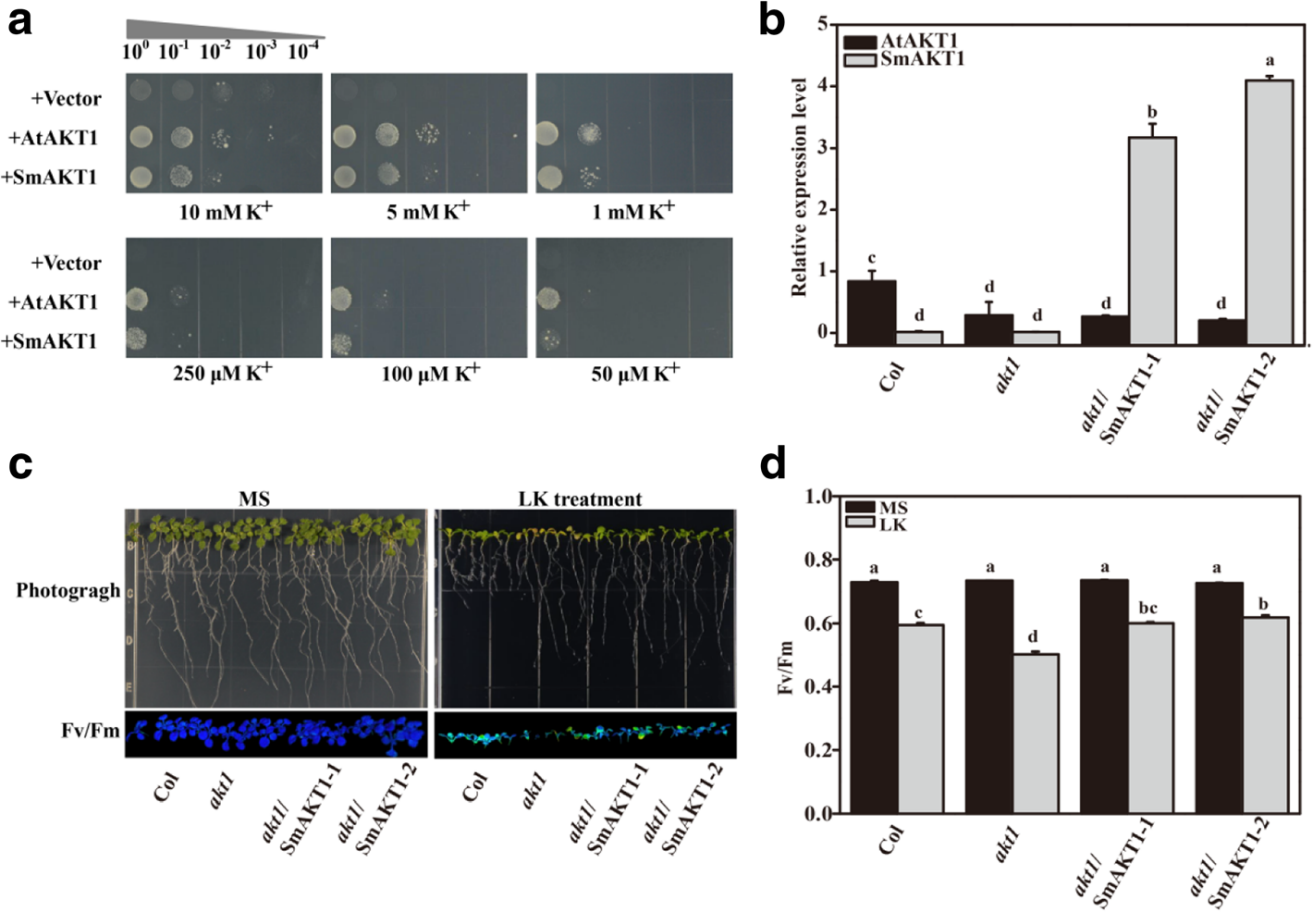
Functional characterization of SmAKT1 in yeast and Arabidopsis under low K^+^ condition. **a** SmAKT1 and AKT1 complement the K^+^ uptake-deficient yeast mutant R5421 on AP medium containing different K^+^ concentrations. **b** Real-time PCR verification of SmAKT1 and AKT1 expression in different plant materials. **c** Phenotype comparison of wild-type Arabidopsis (Col), *akt1* mutant and two complementary lines (*akt1*/SmAKT1–1 and *akt1*/SmAKT1–2) grown on MS and LK (100 mM K^+^) medium for 7 d. **d** Average Fv/Fm values of the whole plant. Bars represent means ± SD of three biological replicates. Columns with different letters indicate significant differences at *P* < 0.05 (Duncan’s test)

Subsequently, the K^+^ transport activity of SmAKT1 was tested in the auxotrophic yeast mutant strain R5421 *(trk1△*, *trk2△*) [50, 65, 66] and Arabidopsis *akt1* mutant [52, 62, 64], respectively. The complementation assays in yeast showed that along with the decline of K^+^ concentration, the growth of R5421 with empty vector was significantly depressed while both SmAKT1 and AtAKT1 could rescue the growth defect of R5421 mutant (Fig. 5a). In addition, the K^+^ deficiency symptoms phenotype of *akt1* mutant was rescued in the two complementary Arabidopsis lines (*akt1*/SmAKT1–1 and *akt1*/SmAKT1–2), which displayed a similar phenotype with wild-type (Col) plants (Fig. 5b-d). These results suggested that SmAKT1 conferred significant K^+^ uptake in yeasts and Arabidopsis under low K^+^ concentrations condition.

**Fig. 6:**
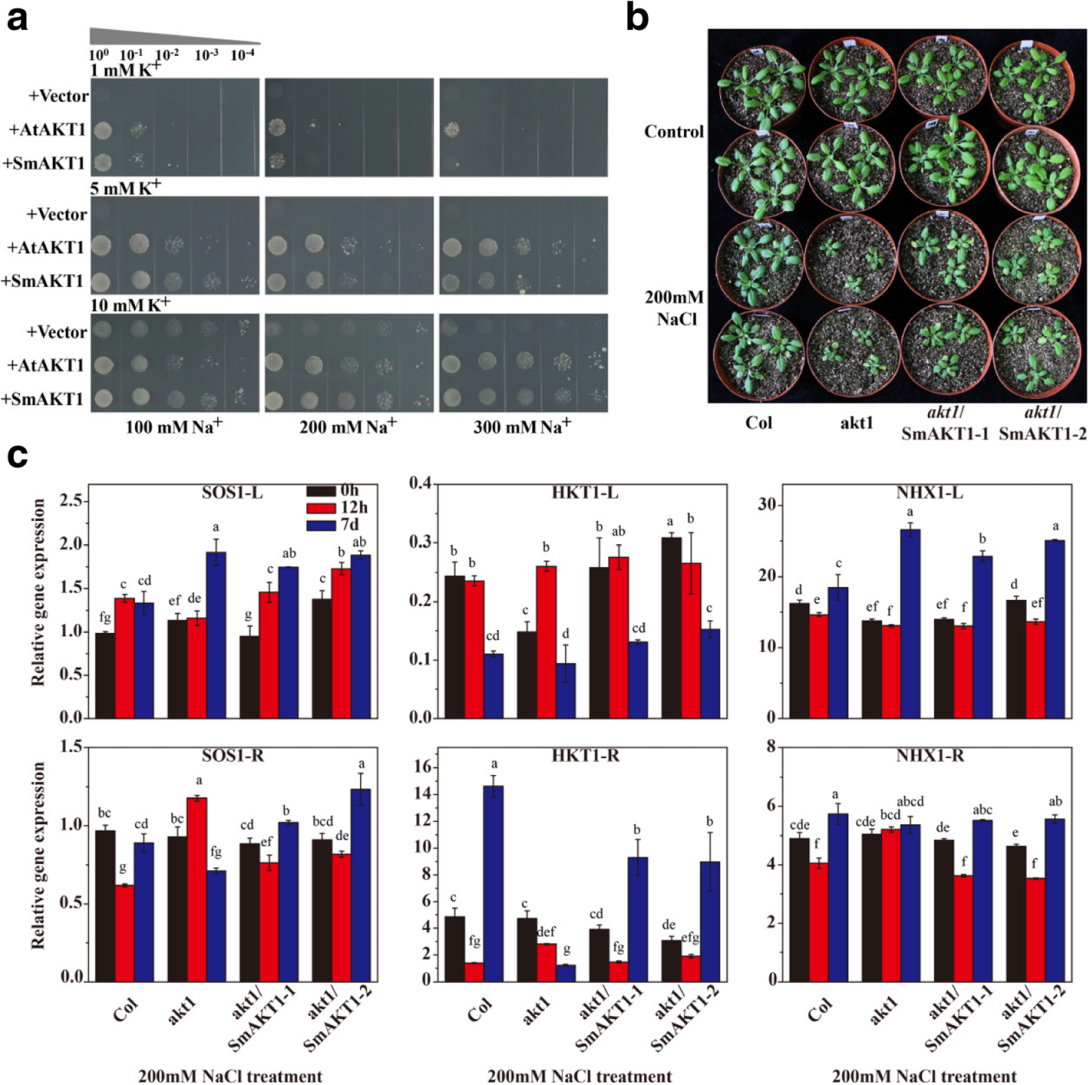
SmAKT1 is involved in response to salt stress in yeast and Arabidopsis under salt condition. **a** Expression of SmAKT1 and AKT1in yeast mutant strain R5421. Yeast cells were plated on AP medium containing various concentrations of Na^+^ (100, 200 and 300 mM) with different K^+^ concentrations (1, 5 and 10 mM). **b** Phenotype comparison of the four Arabidopsis lines after 200 mM NaCl treatment for 0 and 7 days. **c** Real-time quantitative PCR analysis of the expression pattern of SOS1, HKT1 and NHX in the four Arabidopsis lines treated without (control) or with 200 mM NaCl for 12 h and 7 days. Bars represent means ± SD of three biological replicates. Columns with different letters indicate significant differences at P < 0.05 (Duncan’s test)

In addition, the transformed yeasts were plated on AP medium containing 1, 5 or 10 mM KCl in combination with 100, 200 or 300 mM NaCl, and the yeasts expressing *SmAKT1* and *AtAKT1* were able to tolerate higher salt stress than the yeast with empty vector (Fig. 6a). In Arabidopsis, comparing with the WT, the growth of *akt1* mutant was inhibited throughout development but was partly recovered in the two complementary lines under control condition (Fig. 6b). These results indicated that SmAKT1 was involved in responding to salt stress.

In order to further explore the potential molecular mechanisms underlying the above observations in Arabidopsis, the expression patterns of genes related to Na^+^ extrusion and transport were analyzed in the four plants under salt conditions with 200 mM NaCl (Fig. 6c). The expression patterns of *SOS1*, *HKT1* and *NHX1* in the two complementary lines were all the same with WT in both leaves and roots, while it was completely different with the *akt1* mutant, except for *NHX1* in leaves after 200 mM NaCl treatment for 0 h, 12 h and 7 days.

Taken together, we speculated that SmAKT1 could enhance the salt tolerance of plants not only through modulating K^+^ uptake, but also altering Na^+^ exclusion, transport and homeostasis under salt conditions.

## Discussion

### Control of the K^+^ and Na^+^ distribution is critical for salt-tolerance

In this study, the salt-tolerances of two eggplant genotypes were characterized. By comparison, the SS30 was more significantly affected than ST118 in the phenotypic and physiological attributes by salt stress, including PH, cross-cut length of stem, SDW, RDW and the concentration and distribution of Na^+^ and K^+^ (Fig. 1 and Additional file 2). These results were in analogy with previous studies in eggplant [30, 67, 68]. It was well known that K^+^/Na^+^ ratio in leaves is an important indicator to measure the salt-tolerance of plants [22, 23]. Here, the K^+^/Na^+^ ratio in ST118 was significantly higher in leaves but lower in roots compared with the SS30 (Fig. 2b). Although total K^+^ concentration was a bit lower in ST118 than in SS30, higher K^+^_[leaves]_/K^+^_[roots]_ ratio were observed in ST118 than SS30. Conversely, higher total Na^+^ content but lower Na^+^_[leaves]_/Na^+^_[roots]_ were observed in ST118 than SS30 (Fig. 2a and Additional file 2). These results suggested that ST118 preferentially translocated K^+^ from roots to leaves, but restricted Na^+^ accumulation in leaves in order to maintain a higher K^+^/Na^+^ ratios (Fig. 2b). Taken together, we speculated that the distribution mechanism of K^+^ and Na^+^ might be another key factor that determined the different salt-resistance of two eggplant genotypes.

### Effect of salt stress on transcriptome changes in SS30 and ST118

Here, the comparative-transcriptome analysis between SS30 and ST118 was carried out in a way similar to previous studies in Arabidopsis [34], rice [3] and tomato [35]. Consistent with earlier studies in rice [2] and Arabidopsis [55], genotype-specific and organ-specific manners also existed in eggplant in response to salt stress (Fig. 3). Since the expression patterns of common DEGs in the leaves/roots of the two eggplant genotypes were almost same (Additional file 5), the genotype-specific DEGs in ST118 were likely responsible for the higher salt-tolerance.

The expressions of genes encoding 2 NACs, WRKY, MYB and COL transcription factors were found different between SS30 and ST118 (Table 1), which were valuable for further investigation in eggplants. Some studies have reported that the members of NAC [10, 11], MYB [13–15] and WRKY [16, 17] were involved in response to elevated external salinity. However, few studies on the function of COL family members in salinity tolerance have been reported so far. Although JH Min, et al. [69] reported that the AtCOL4-overexpressing plants were more tolerant to salt stress than the wild-type, most researches of the COL genes family focused on exploring its function on the flowering time of plants, such as OsCOL10 [70], OsCOL9 [71] and GhCOL1 [72]. In addition, previous studies reported that BTB/TAZ played an essential role during gametogenesis, and probably throughout plant development [73]. Recently, Q Zhao, et al. [74] reported that MdBT1/2 (a BTB/TAZ protein) interact with MdCUL3 to bridge the formation of the MdBTs^MdCUL3^ complex, which negatively modulates the degradation of the MdbHLH104 protein in response to changes in Fe status to maintain iron homeostasis in plants. And V Araus, et al. [75] reported that BT2 was the most central and connected gene in the nitrogen use efficiency (NUE) network in Arabidopsis and rice. Taken together, we thought that the 6 TFs are good candidates for further investigation of their role in salinity tolerance.

### Candidate genes associated with K^+^ and Na^+^ homeostasis

Maintaining ion homeostasis is one of the key determinants for the plants survival under salt stress. The finding in this work that the Na^+^_[leaves]_ /Na^+^_[roots]_ increased less in ST118 than in SS30 along with salt treatment, indicating that ST118 may possess a mechanism to restrict the accumulation of Na^+^ in the leaves. The ‘salt overly sensitive’ (SOS) signaling pathway, including SOS1, SOS2 and SOS3 genes, has been proven to be important for plant salt tolerance [76, 77]. Among them, *SOS1* was well known to be expressed in root epidermal cells and xylem parenchyma cells and was involved in extruding Na^+^ into the external medium and loading Na^+^ into the xylem for long-distance transport to leaves [61, 78, 79]. However, the *SOS1* were expressed constitutively at higher levels in the roots of both eggplant genotypes. Strikingly, *SOS1* was significantly up-regulated in the leaves of ST118 but was slightly down-regulated in SS30. Previous studies have been reported that *SOS1* is also expressed in the xylem parenchyma in leaves but where its function is unclear so far. JK Zhu [18] speculated that the function of SOS1 in leaves may function to extrude Na^+^ from the xylem parenchyma cells into the apoplastic space of mesophyll cells.

Except SOS1, seven genes encoding K^+^ transporters or K^+^ channel proteins were identified in leaves to be up-regulated in response to salt stress (Table 2). It is worth noting that the genes encoding KAT1 and AKT1 were significantly up-regulated only in ST118. These results could partially explain the higher K^+^_[leaves]_ /K^+^_[roots]_ ratio in ST118 than in SS30 under salt stress. Similar with *SOS1*, *AKT1* were expressed constitutively at higher levels in the roots of both two eggplant genotypes (Table 2). AKT1 was the first inward-rectifying K^+^ channel identified in Arabidopsis by functional complementation of yeast mutant strains defective in K^+^ transport system [80]. Moreover, a model of K^+^ uptake regulated by AKT1 in Arabidopsis and *Oryza sativa* under low-K^+^ conditions was proposed [50, 52]. In addition, previous studies showed that the osmotic- and drought-tolerance of rice could be enhanced by overexpression of *OsAKT1* [81]. In fact, K^+^ deficiency would be accompanied by excessive accumulation of Na^+^ under salt stress. However, extensive researches were directed to the genes related to the influx, extrusion and accumulation of Na^+^ to improve K^+^/Na^+^ ratio in plants. Relatively limited studies focused on investigation of the AKT1 roles in maintaining K^+^ and Na^+^ homeostasis in plants under salt stress, especially in eggplants [82–84].

### SmAKT1 is not only involved in modulating K^+^ uptake, but also in altering Na^+^ exclusion, transportation and homeostasis in Arabidopsis under salt conditions

In this study, more genes related to K^+^ uptake were identified as DEGs than those related to Na^+^ regulation (Table 2). Subsequently, the complementation assays in both yeast and Arabidopsis *akt1* mutants demonstrated that SmAKT1 was involved in response to both low-K^+^ condition (Fig. 5) and salt conditions (Fig. 6a, b). Given the phenotype of K^+^ concentration and distribution in ST118 under salt stress, we speculated that SmAKT1 not only mediates K^+^ uptake in roots, but is also essential for maintaining long-distance transport and homeostasis of K^+^ in eggplants, which is similar with Arabidopsis [52] and *Z. xanthoxylum* [83]. In addition, the expression patterns of *SOS1*, *HKT1* and *NHX1*, known as Na^+^ uptake and transport genes, were significantly changed in the Arabidopsis *akt1* mutants and recovered in the complementary lines, when compared with the wild type under salt stress.

As described above, the functions of SOS1 in roots were Na^+^ extrusion and Na^+^ upload into the xylem [18, 78, 79]. However, the coordination mechanism of these two roles is not well understood. The other important Na^+^ transporter is HKT1, which acts in the retrieval of Na^+^ from the xylem to restrict the Na^+^ amount in the transpiration stream in roots [85] and uploading Na^+^ into the phloem for recirculation back to roots from the leaves [86]. In this work, the transcription level of *SOS1* was significantly increased in roots but no change was observed in leaves of *akt1* mutants after short-term salt treatment (12 h) (Fig. 6c). However, the transcription level of *HKT1* was significantly decreased in roots but was increased in leaves at the same time (Fig. 6c). Taken together, we speculated that the *akt1* mutant transported Na^+^ into leaves by SOS1, while the leaves restrict Na^+^ accumulation in leaves by the function of HKT1. And these opposing works by two different genes might be an important reason for its intolerance to salt stress. In contrast to the *akt1* mutant, the wild type and the two complementary lines might have developed a mechanism to avoid Na^+^ from entering into leaves and to transfer Na^+^ into the apoplastic space of mesophyll cells as soon as possible. In addition, after being exposed to prolonged salt stress (7d), the wild type and the two complementary lines unload the Na^+^ from xylem by upregulating the expression of *HKT1* and extrude it to soil solution by upregulating the expression of *SOS1*.

As for NHX1, it seems to be not closely to AKT1, and the function of NHX1 in plant leaves has been well studied while it was only partly understood in roots, which could transport the excessive Na^+^ to vacuole. Here, the *NHX1* was down-regulated in roots of the three tolerant Arabidopsis lines at 12 h but up-regulated at 7 days (Fig. 6c). It could be explained as that the vacuole was the ultimate storage space for additional Na^+^.

Taken together, our results suggested that SmAKT1 is an important determinant for maintaining K^+^ and Na^+^ homeostasis in eggplant under salt stress, since it not only mediates K^+^ uptake, but also modulates Na^+^ uptake and transport systems.

## Conclusion

In order to grow on saline soils, plants developed coordinated physiological traits throughout the lifecycle, among which the K^+^ and Na^+^ homeostasis is a key determinant to evaluate salt-tolerance. Here, comparative analysis of transcript levels in response to salt stress between salt-sensitive and salt-tolerant eggplant genotypes provided insights into key candidate genes related to salinity tolerance. The transcriptomic differences between SS30 and ST118 indicated the diversity of approaches to resist the challenge of salt stress. Further, the differently expressed TFs and ion transport genes were selectively analyzed, and the complementation assays demonstrated that SmAKT1 is an important regulator under salt conditions. Objectively, it also suggested that the other TFs and K^+^ transport genes were also worth further investigation for their functions in salinity tolerance. These data provides a foundation for elucidating the molecular networks underlying salt tolerance in eggplants.

## Additional files

Additional file 1: **Table S1.** List of primers sequences used in this study. (DOCX 32 kb)

Additional file 2: **Figure S1.** The K^+^ (**a**) and Na^+^ (**b**) content in leaves and roots of two eggplant genotypes along with 200 mM NaCl treatment. DW represents dry weight. Three replicates were used in each time point, with three seedlings per replicate. Bars represent means ± SD of three biological replicates. Duncan’s Multiple Range test (*P < 0.05 and **P < 0.01) was used to analyze statistical significance. (DOCX 391 kb)

Additional file 3: **Table S2.** Summary statistics of sequencing and assembly. Tissue: The tissue of eggplant seedling; Samples: Sample names; Total Clean Reads(Mb): The reads amount after filtering, Unit: Mb; Clean Reads Ratio(%): The ratio of the amount of filtered clean reads; Total Mapping Ratio: The percentage of mapped reads; Uniquely Mapping Ratio: The percentage of uniquely mapped reads (%); Expressed Gene No.: The amount of expressed genes; SS represents salt sensitive eggplant SS30; ST represents salt tolerant eggplant ST118; 0 h and 12 h represent the time after NaCl treatment; L: leaves; R: Roots. (DOCX 40 kb)

Additional file 4: **Figure S2.** Validation of RNA-seq data in leaves (**a**) and roots (**b**) using qRT-PCR. (DOCX 1923 kb)

Additional file 5: **Figure S3.** Four-way Venn diagram indicating the number of salt-up-regulated and -down-regulated genes found exclusively in the leaves (**a**) and roots (**b**) of two eggplant genotypes in the comparison between salt-stressed and non-stress treatments. (DOCX 468 kb)

Additional file 6: **Figure S4.** GO classification of up- and down-regulated genes in leaves or roots of SS30 or ST118. (DOCX 1470 kb)

Additional file 7: **Figure S5.** Overview the salt-up- or down-regulated TFs in the leaves and roots of both two eggplant genotypes at a level of ≥2-fold and adjusted *P*-value ≤0.001. (DOCX 3480 kb)

Additional file 8: **Figure S6.** Phylogenetic relationships of the two SmAKT1s with AKT1 from other species. Protein sequences of AKT1 were analyzed using MEGA7.0 and the Neighbor-Joining method with 1000 bootstrap replicates. (DOCX 258 kb)

Additional file 9: **Figure S7.** The conserved domains in across AKT1 proteins. The overall height of each stack indicates the conservation of the sequence at that position, whereas the height of letters within each stack represents the relative frequency of the corresponding amino acid. (DOCX 1146 kb)

## Abbreviations

FAO: Food and Agriculture Organization of the United Nations
SS30-L/R-Spe: The DEGs specifically identified in the leaves/roots of SS30
ST118-L/R-Spe: The DEGs specifically identified in the leaves/roots of ST118
STIDB: The Stress Responsive Transcription Factor Database
ST-SS-L/R-inter: The intersection of DEGs identified in the leaves/roots of both SS30 and ST118
