# Supplementary material for: Comparative transcriptome analysis reveals K+ transporter gene contributing to salt tolerance in eggplant
Source: BMC Plant Biol. 2019 Feb 11;19:67. doi: 10.1186/s12870-019-1663-8 (PMC6371450; doi:10.1186/s12870-019-1663-8)
Supplement: Supplementary file 1 — Table S1. List of primers sequences used in this study. (DOCX 32 kb) [file 12870_2019_1663_MOESM1_ESM.docx]

**Additional files 1: Table S1.** List of primers sequences used in this study

| **Gene code** | **Gene name** | **Forward primer (5’-3’)** | **Reverse primer (5’-3’)** |
| --- | --- | --- | --- |
| **For real-time qPCR** |  |  |  |
| SOS1 | Sme2.5_05879.1_g00004.1 | GGGAGATCTCGGGAATGGAAT | TGGAGAATGCGCCCTCAAA |
| C2C2-CO-like | Sme2.5_25982.1_g00001.1 | GCGTTTACTGCGACCAGGACTA | GTGATTGTGTTCGGTGGAATCC |
| MYB | Sme2.5_02214.1_g00006.1 | GGAAAACGCTGGTGCACACA | AGCAGTGAGGACCCTATCCAAGA |
| WRKY | Sme2.5_01585.1_g00006.1 | ACCGACCTTCTTGCTTCTGATGA | GGAACTCCAGAACCAGTCCTCTCT |
| K^+^ channel protein | Sme2.5_00325.1_g00013.1 | TACCGTGCCACCCTGTTTTG | ATAGGACTCTGAAAGCGGTGGAGTA |
| bHLH | Sme2.5_00036.1_g00016.1 | CTTTGATGAGTCTTCGCGTAGCA | GTTTTCCGAGCCACCACCAT |
| WRKY | Sme2.5_00556.1_g00019.1 | ACTCCTTCCGATCAAACCTCAATG | GGCATGTCGGTGCAAATGA |
| AKT1-like | Sme2.5_09079.1_g00001.1 | TGAATATCAGGGCCACGAAGAG | AGGAGGACGGACATTGGTTTCTTT |
| AKT1 | Sme2.5_00439.1_g00001.1 | ATGCTAGCTCGCGGTAGAATGG | TCAAGACCGCGCTTCAACA |
| Smactin | Sme2.5_01462.1_g00018.1 | GTCGGAATGGGACAGAAGGATG | GTGCCTCAGTCAGGAGAACAGGGT |
| AtAKT1 | AT2G26650.1 | TGCACTCCCAAAGGCCATTA | GGTCATTGGATACTCCTCGAAACA |
| AtSOS1 | AT2G01980.1 | CTTGTGAAGGTCACGTTTCCGTAT | CACCAAGCTCCTTTAGCAAAGCA |
| AtHKT1 | AT4G10310.1 | GTGACGGTGCTAGGGTTTCTGAT | CCAACTTCTCGTACGAACTCATTCC |
| AtNHX1 | AT5G27150.1 | CACACAGATGTACGCGGGAATG | GCTTATGAGTGGTTTGGTCAGCAT |
| AtACT2 | AT3G18780.1 | CTTGCACCAAGCAGCATGAA | CCACCGATCCAGACACTGTACTT |
| **For expression assays** |  |  |  |
| PHB-SmAKT1 | Sme2.5_00439.1_g00001.1 | TCTCTCTCTCAAGCTTATGGGGGAAAATAGAGGACTTGG | TGCAGCTCGAGGATCCCAATTCTGGTGATTTATCTTCATGG |
| pYES2-AtAKT1 | AT2G26650.1 | AGGGAATATTAAGCTTATGAGAGGAGGGGCTTTGTTATG | GATATCTGCAGAATTCTTAAGAATCAGTTGCAAAGATGAGATG |
| pYES2-SmAKT1 | Sme2.5_00439.1_g00001.1 | AGGGAATATTAAGCTTATGGGGGAAAATAGAGGACTTGG | GATATCTGCAGAATTCTTACAATTCTGGTGATTTATCTTCATGG |
