# Supplementary material for: Comparative transcriptome analysis reveals K+ transporter gene contributing to salt tolerance in eggplant
Source: BMC Plant Biol. 2019 Feb 11;19:67. doi: 10.1186/s12870-019-1663-8 (PMC6371450; doi:10.1186/s12870-019-1663-8)
Supplement: Supplementary file 2 — Figure S1. The K+ (a) and Na+ (b) content in leaves and roots of two eggplant genotypes along with 200 mM NaCl treatment. DW represents dry weight. Three replicates were used in each time point, with three seedlings per replicate. Bars represent means ± SD of three biological replicates. Duncan’s Multiple Range test (*P < 0.05 and **P < 0.01) was used to analyze statistical significance. (DOCX 391 kb) [file 12870_2019_1663_MOESM2_ESM.docx]

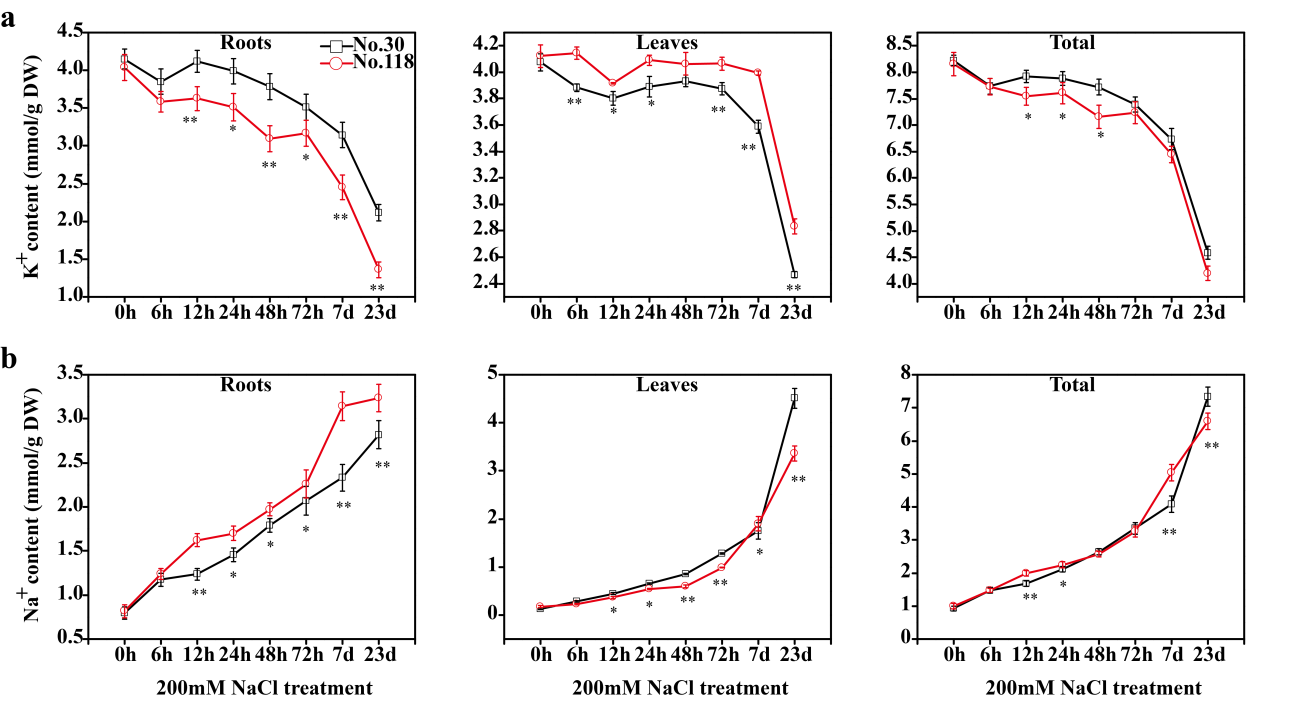


**Additional file 2: Figure S1.** The K^+^ (**a**) and Na^+^ (**b**) content in leaves and roots of two eggplant genotypes along with 200 mM NaCl treatment. DW represents dry weight. Three replicates were used in each time point, with three seedlings per replicate. Bars represent means ± SD of three biological replicates. Duncan’s Multiple Range test (*P < 0.05 and **P < 0.01) was used to analyze statistical significance.
