# Supplementary material for: Comparative transcriptome analysis reveals K+ transporter gene contributing to salt tolerance in eggplant
Source: BMC Plant Biol. 2019 Feb 11;19:67. doi: 10.1186/s12870-019-1663-8 (PMC6371450; doi:10.1186/s12870-019-1663-8)
Supplement: Supplementary file 3 — Table S2. Summary statistics of sequencing and assembly. Tissue: The tissue of eggplant seedling; Samples: Sample names; Total Clean Reads(Mb): The reads amount after filtering, Unit: Mb; Clean Reads Ratio(%): The ratio of the amount of filtered clean reads; Total Mapping Ratio: The percentage of mapped reads; Uniquely Mapping Ratio: The percentage of uniquely mapped reads (%); Expressed Gene No.: The amount of expressed genes; SS represents salt sensitive eggplant SS30; ST represents salt tolerant eggplant ST118; 0 h and 12 h represent the time after NaCl treatment; L: leaves; R: Roots. (DOCX 40 kb) [file 12870_2019_1663_MOESM3_ESM.docx]

**Additional files 3: Table S2.** Summary statistics of sequencing and assembly**.** Tissue: The tissue of eggplant seedling; Samples: Sample names; Total Clean Reads(Mb): The reads amount after filtering, Unit: Mb; Clean Reads Ratio(%): The ratio of the amount of filtered clean reads; Total Mapping Ratio: The percentage of mapped reads; Uniquely Mapping Ratio: The percentage of uniquely mapped reads (%); Expressed Gene No.: The amount of expressed genes; SS represents salt sensitive eggplant SS30; ST represents salt tolerant eggplant ST118; 0h and 12 h represent the time after NaCl treatment; L: leaves; R: Roots.

| Tissue | Sample | Total Clean Reads (Mb) | Clean Reads Ratio (%) | Total Mapping Ratio | Uniquely Mapping Ratio | Total Gene No. |
| --- | --- | --- | --- | --- | --- | --- |
| Leaves | SS_0h_L1 | 24.12 | 99.92 | 65.75 | 55.1 | 34961 |
|  | SS_0h_L2 | 24.12 | 99.92 | 65.79 | 55.03 | 35469 |
|  | SS_0h_L3 | 24.12 | 99.94 | 65.86 | 55.23 | 35545 |
|  | SS_12h_L1 | 24.12 | 99.91 | 61.22 | 51.21 | 34016 |
|  | SS_12h_L2 | 24.11 | 99.87 | 63.49 | 53.03 | 35461 |
|  | SS_12h_L3 | 24.12 | 99.91 | 63.77 | 53.23 | 35004 |
|  | ST_0h_L1 | 24.12 | 99.92 | 66.54 | 54.92 | 32784 |
|  | ST_0h_L2 | 24.11 | 99.91 | 66.69 | 55.1 | 32054 |
|  | ST_0h_L3 | 24.12 | 99.93 | 66.95 | 55.44 | 33951 |
|  | ST_12h_L1 | 24.11 | 99.9 | 66 | 54.91 | 34386 |
|  | ST_12h_L2 | 24.1 | 99.86 | 65.49 | 53.79 | 33477 |
|  | ST_12h_L3 | 24.11 | 99.89 | 65.65 | 54.27 | 33974 |
| Roots | SS_0h_R1 | 24.05 | 99.65 | 59.66 | 50.74 | 34564 |
|  | SS_0h_R2 | 24.11 | 99.9 | 57.5 | 48.83 | 33983 |
|  | SS_0h_R3 | 24.11 | 99.88 | 55.55 | 47.13 | 33486 |
|  | SS_12h_R1 | 24.1 | 99.85 | 58.88 | 50.09 | 36014 |
|  | SS_12h_R2 | 24.1 | 99.86 | 55.99 | 47.44 | 33638 |
|  | SS_12h_R3 | 24.1 | 99.86 | 57.55 | 48.81 | 34131 |
|  | ST_0h_R1 | 24.1 | 99.84 | 62.01 | 52.8 | 34200 |
|  | ST_0h_R2 | 24.1 | 99.83 | 58.6 | 49.6 | 32089 |
|  | ST_0h_R3 | 20.34 | 99.37 | 57.25 | 48.41 | 31157 |
|  | ST_12h_R1 | 24.09 | 99.81 | 60.55 | 51.8 | 35119 |
|  | ST_12h_R2 | 24.03 | 99.56 | 57.95 | 49.1 | 32316 |
|  | ST_12h_R3 | 24.05 | 99.62 | 60.09 | 51.11 | 33155 |
