# Supplementary figures and images for: Comparative transcriptome analysis reveals K+ transporter gene contributing to salt tolerance in eggplant
Source: BMC Plant Biol. 2019 Feb 11;19:67. doi: 10.1186/s12870-019-1663-8 (PMC6371450; doi:10.1186/s12870-019-1663-8)

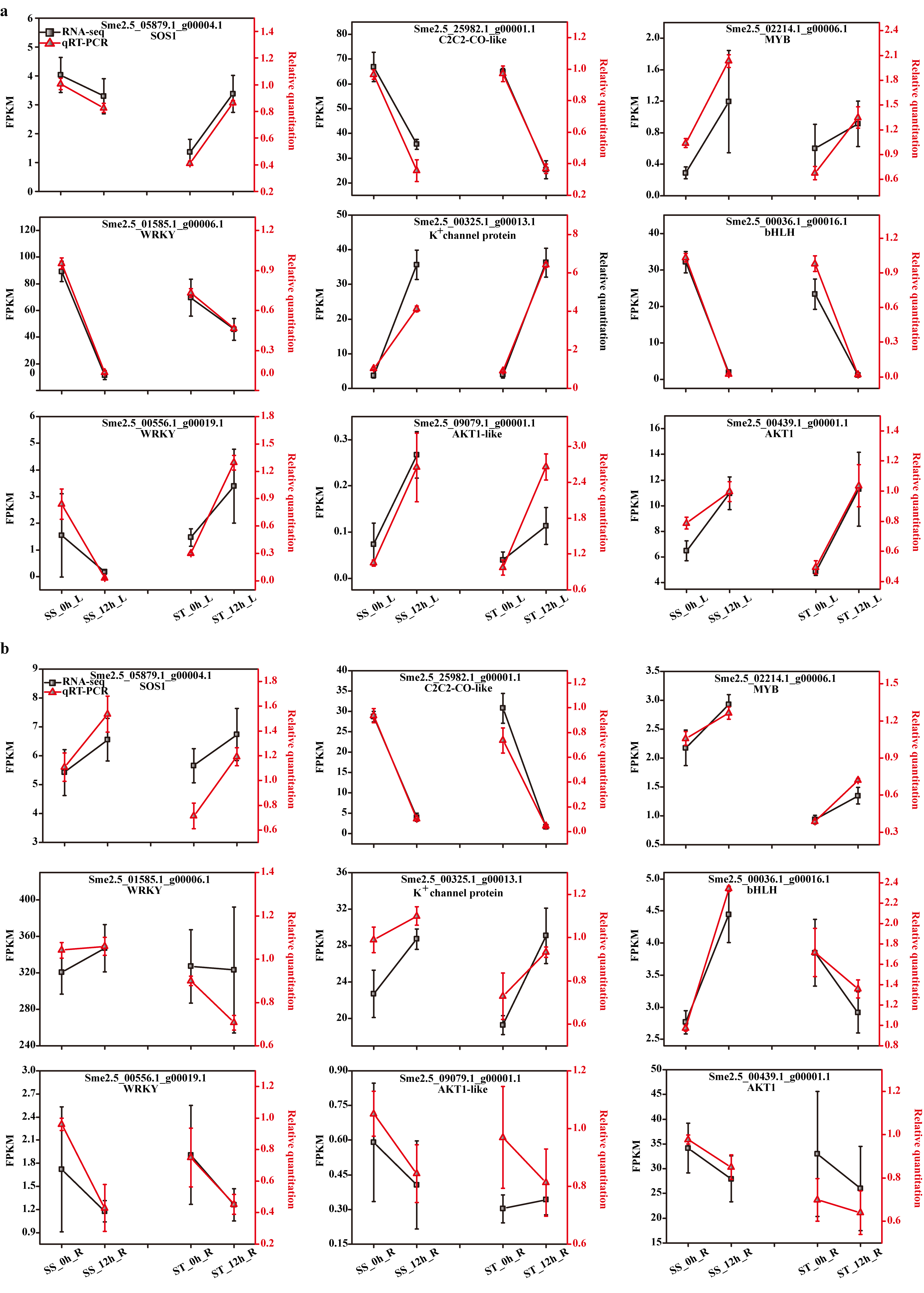


**Additional file 4: Figure S2.** Validation of RNA-seq data in leaves (**a**) and roots (**b**) using qRT-PCR.

Supplement: Supplementary file 4 — Figure S2. Validation of RNA-seq data in leaves (a) and roots (b) using qRT-PCR. (DOCX 1923 kb) [file 12870_2019_1663_MOESM4_ESM.docx]
