# Supplementary material for: Comparative transcriptome analysis reveals K+ transporter gene contributing to salt tolerance in eggplant
Source: BMC Plant Biol. 2019 Feb 11;19:67. doi: 10.1186/s12870-019-1663-8 (PMC6371450; doi:10.1186/s12870-019-1663-8)
Supplement: Supplementary file 5 — Figure S3. Four-way Venn diagram indicating the number of salt-up-regulated and -down-regulated genes found exclusively in the leaves (a) and roots (b) of two eggplant genotypes in the comparison between salt-stressed and non-stress treatments. (DOCX 468 kb) [file 12870_2019_1663_MOESM5_ESM.docx]

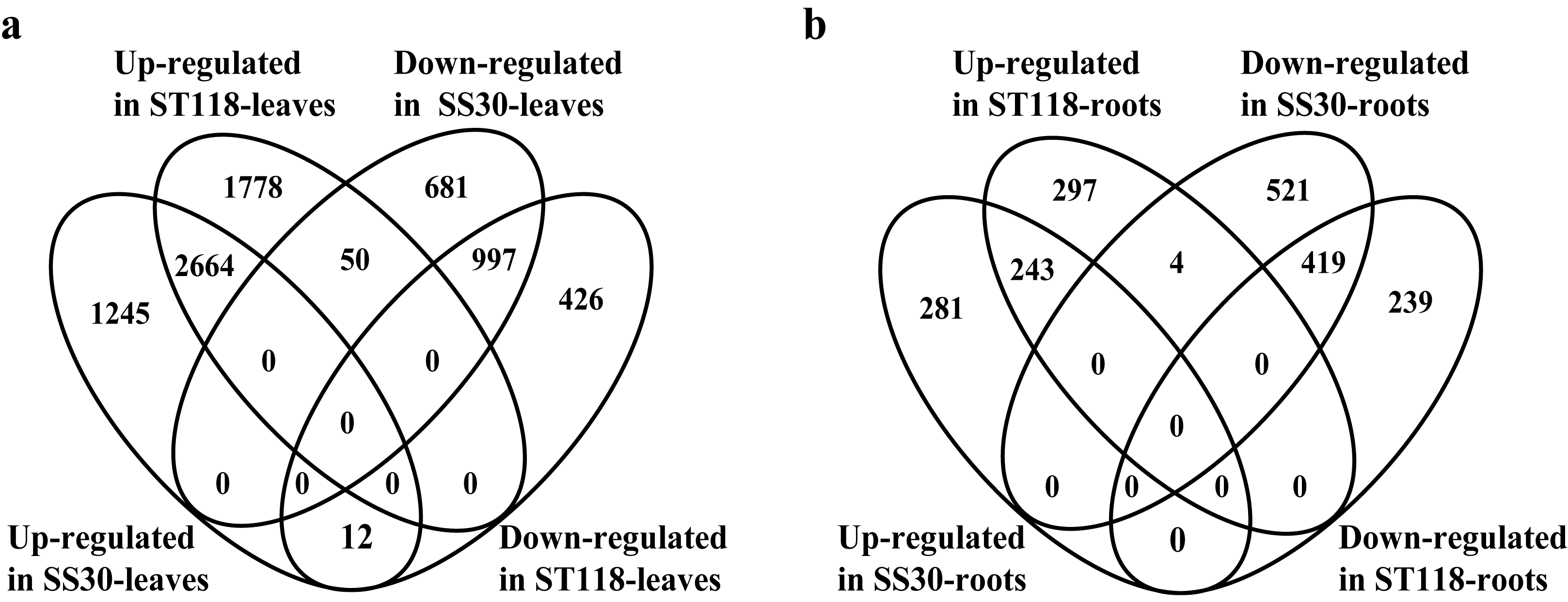


**Additional file 5: Figure S3.** Four-way Venn diagram indicating the number of salt-up-regulated and -down-regulated genes found exclusively in the leaves (**a**) and roots (**b**) of two eggplant genotypes in the comparison between salt-stressed and non-stress treatments.
