# Supplementary material for: Comparative transcriptome analysis reveals K+ transporter gene contributing to salt tolerance in eggplant
Source: BMC Plant Biol. 2019 Feb 11;19:67. doi: 10.1186/s12870-019-1663-8 (PMC6371450; doi:10.1186/s12870-019-1663-8)
Supplement: Supplementary file 6 — Figure S4. GO classification of up- and down-regulated genes in leaves or roots of SS30 or ST118. (DOCX 1470 kb) [file 12870_2019_1663_MOESM6_ESM.docx]

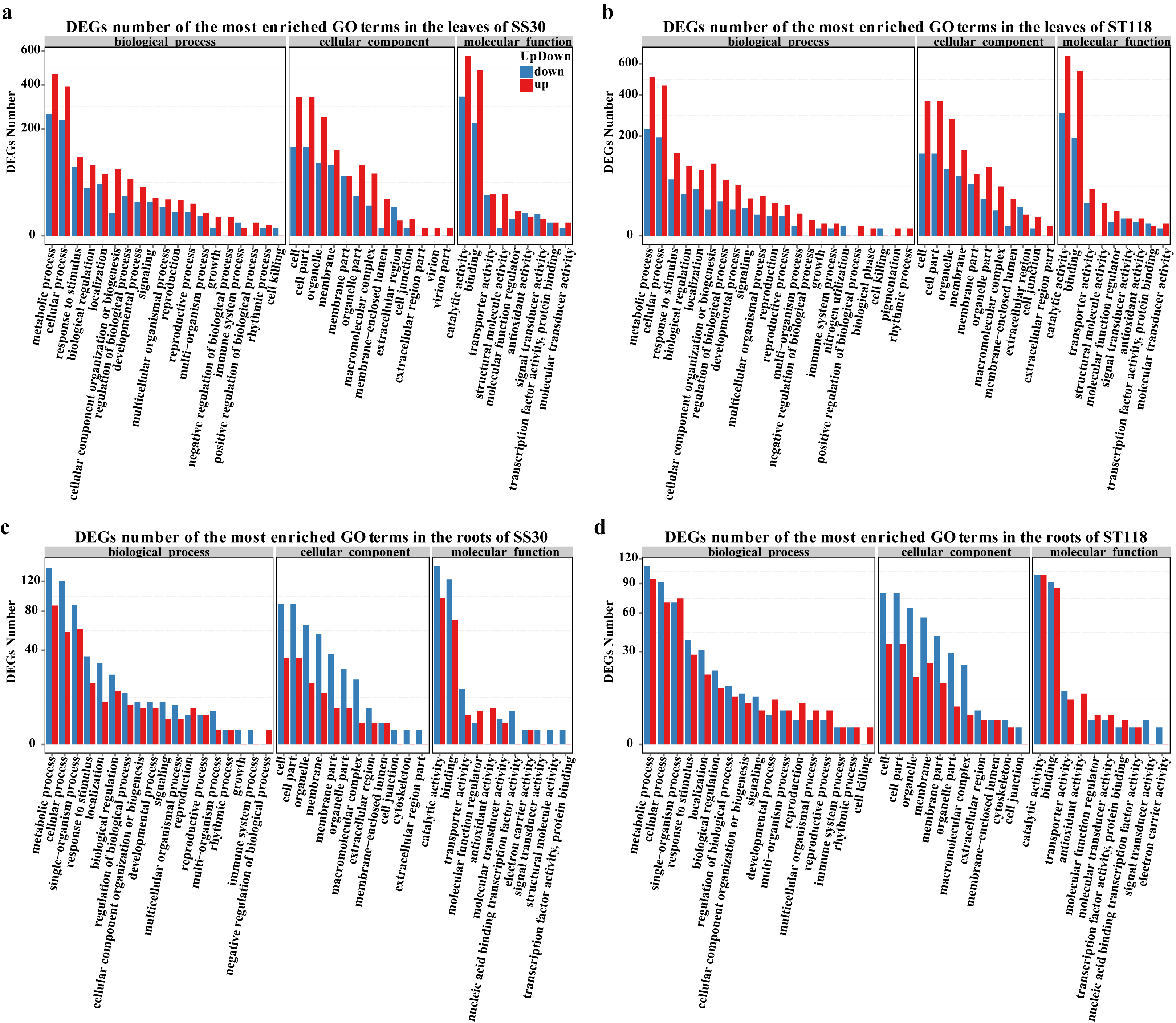


**Additional file 6: Figure S4.** GO classification of up- and down-regulated genes in leaves or roots of SS30 or ST118.
