# Supplementary material for: Comparative transcriptome analysis reveals K+ transporter gene contributing to salt tolerance in eggplant
Source: BMC Plant Biol. 2019 Feb 11;19:67. doi: 10.1186/s12870-019-1663-8 (PMC6371450; doi:10.1186/s12870-019-1663-8)
Supplement: Supplementary file 7 — Figure S5. Overview the salt-up- or down-regulated TFs in the leaves and roots of both two eggplant genotypes at a level of ≥2-fold and adjusted P-value ≤0.001. (DOCX 3480 kb) [file 12870_2019_1663_MOESM7_ESM.docx]

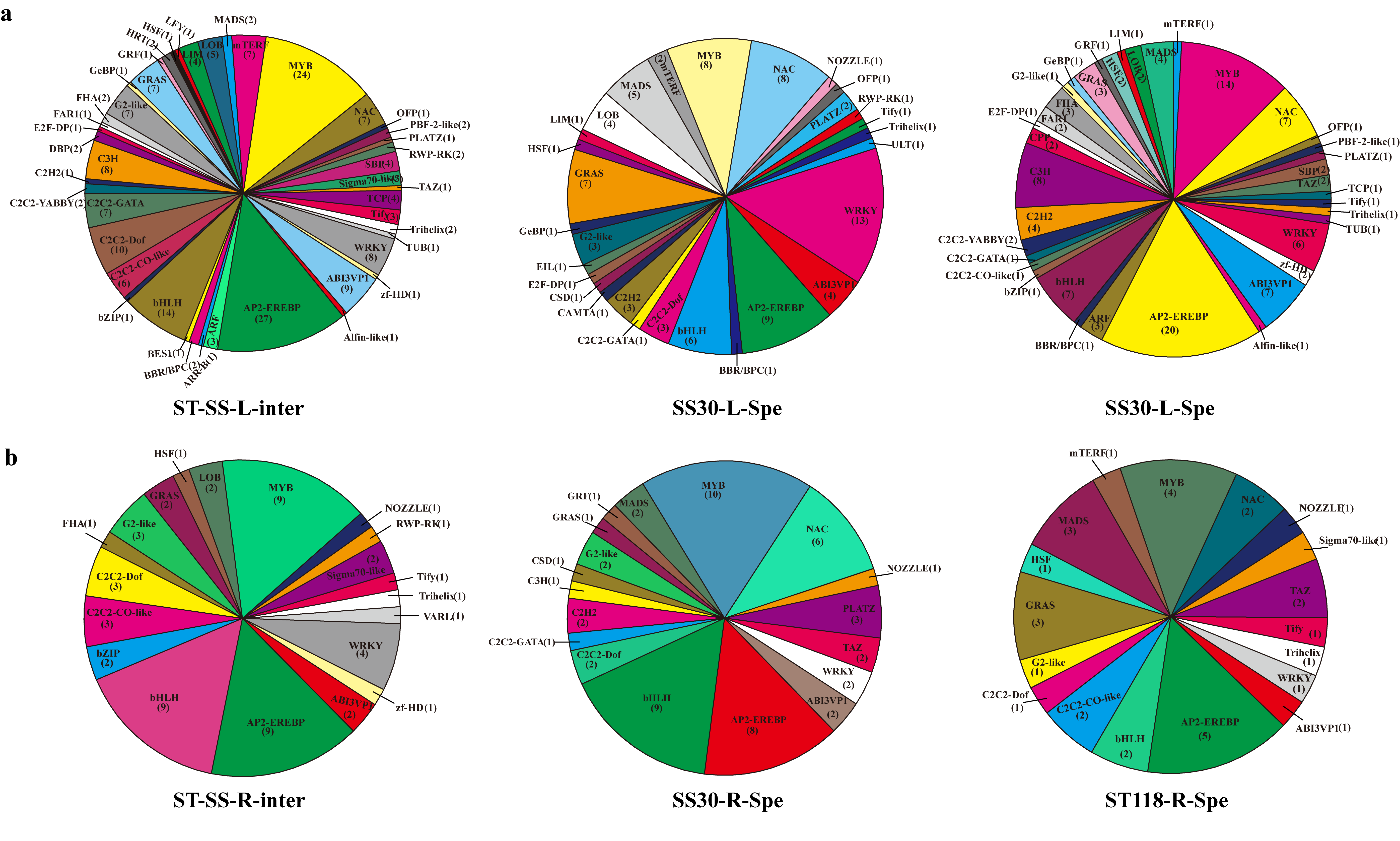


**Additional file 7: Figure S5.** Overview the salt-up- or down-regulated TFs in the leaves and roots of both two eggplant genotypes at a level of ≥ 2-fold and adjusted P-value ≤ 0.001.
