# Supplementary material for: Comparative transcriptome analysis reveals K+ transporter gene contributing to salt tolerance in eggplant
Source: BMC Plant Biol. 2019 Feb 11;19:67. doi: 10.1186/s12870-019-1663-8 (PMC6371450; doi:10.1186/s12870-019-1663-8)
Supplement: Supplementary file 8 — Figure S6. Phylogenetic relationships of the two SmAKT1s with AKT1 from other species. Protein sequences of AKT1 were analyzed using MEGA7.0 and the Neighbor-Joining method with 1000 bootstrap replicates. (DOCX 258 kb) [file 12870_2019_1663_MOESM8_ESM.docx]

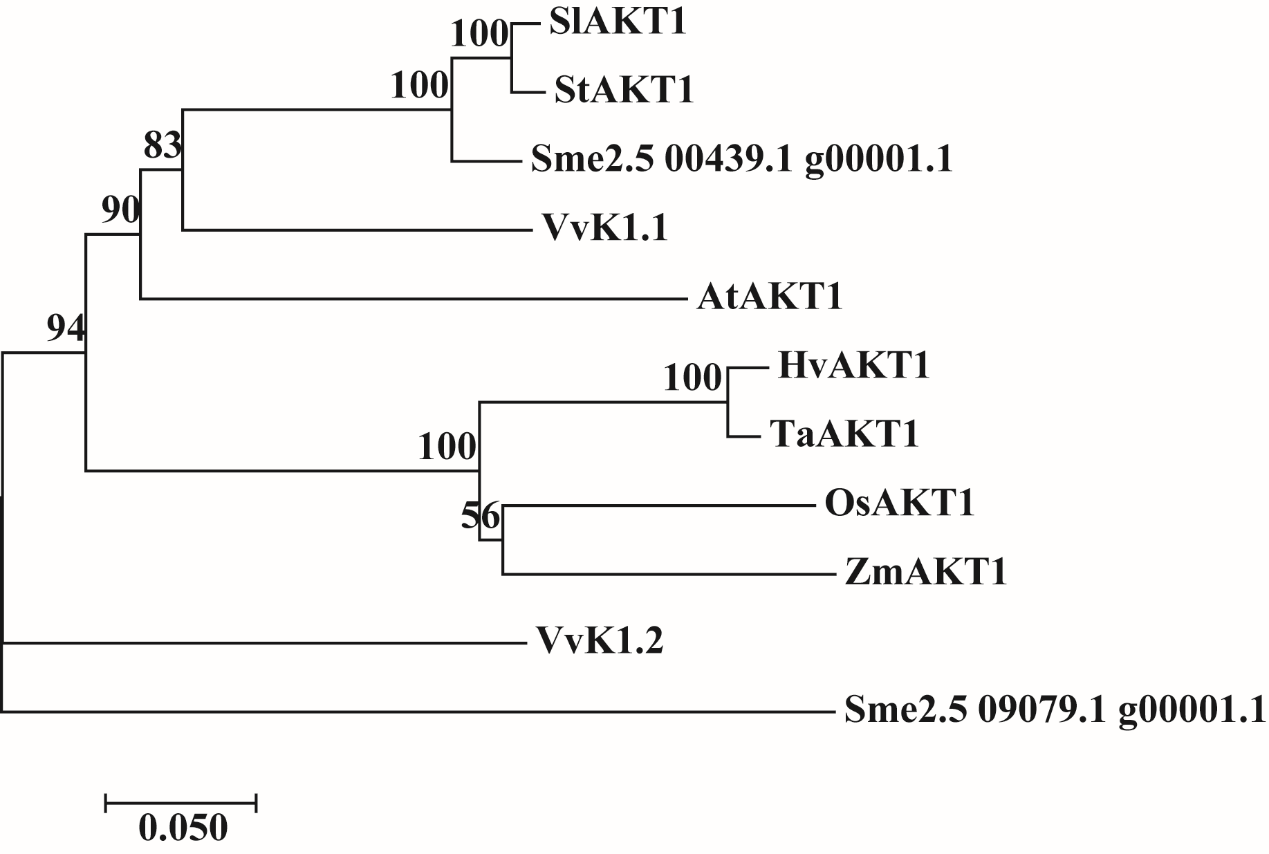


**Additional file 8: Figure S6.** Phylogenetic relationships of the two SmAKT1s with AKT1s from other species. Protein sequences of AKT1 were analyzed using MEGA7.0 and the Neighbor-Joining method with 1000 bootstrap replicates.
