# Supplementary material for: Comparative transcriptome analysis reveals K+ transporter gene contributing to salt tolerance in eggplant
Source: BMC Plant Biol. 2019 Feb 11;19:67. doi: 10.1186/s12870-019-1663-8 (PMC6371450; doi:10.1186/s12870-019-1663-8)
Supplement: Supplementary file 9 — Figure S7. The conserved domains in across AKT1 proteins. The overall height of each stack indicates the conservation of the sequence at that position, whereas the height of letters within each stack represents the relative frequency of the corresponding amino acid. (DOCX 1146 kb) [file 12870_2019_1663_MOESM9_ESM.docx]

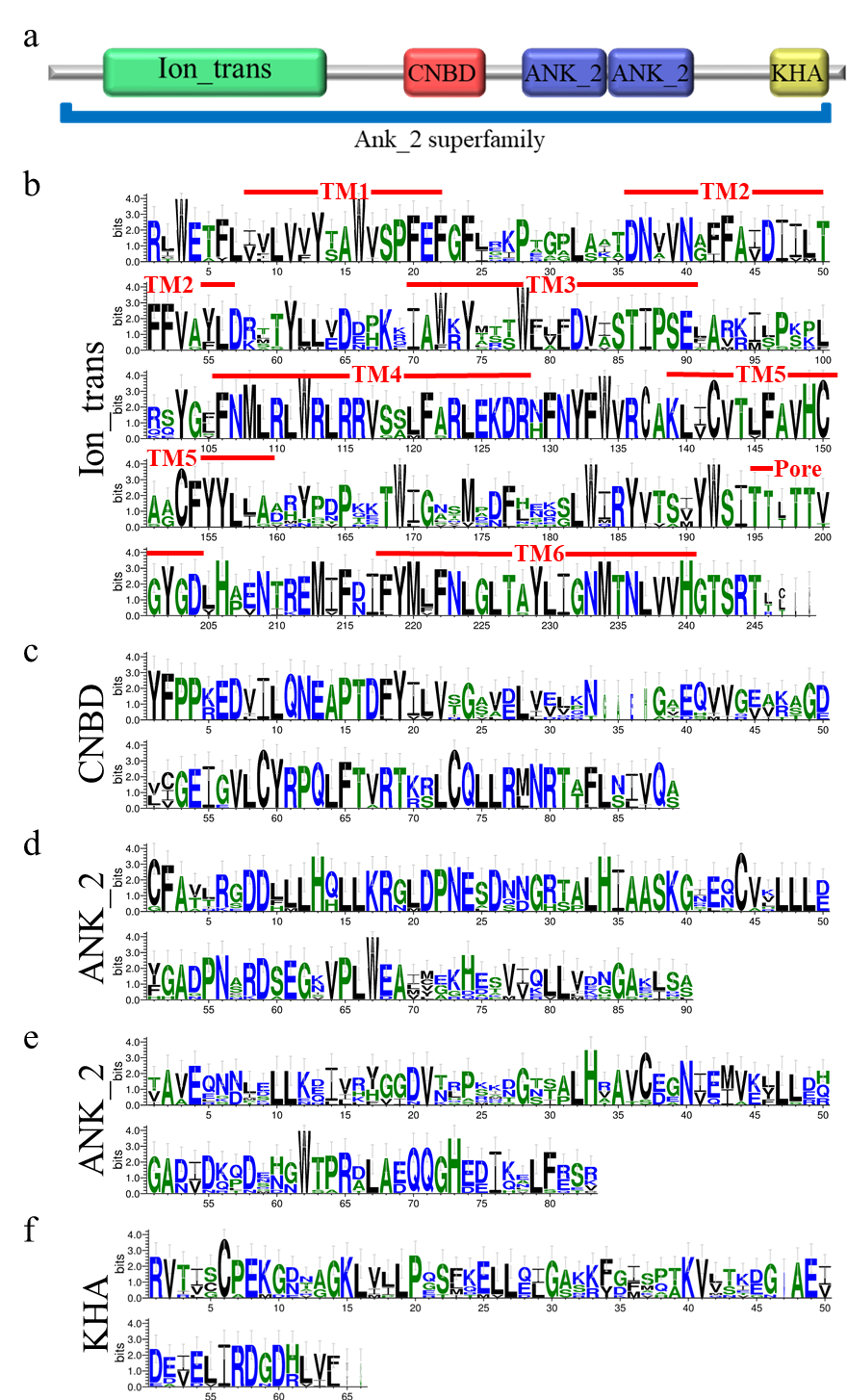


**Additional file 9: Figure S7.** The conserved domains in across AKT1 proteins. The overall height of each stack indicates the conservation of the sequence at that position, whereas the height of letters within each stack represents the relative frequency of the corresponding amino acid.
